# Insights into the Mechanism and Catalysis of Peptide Thioester Synthesis by Alkylselenols Provide a New Tool for Chemical Protein Synthesis

**DOI:** 10.3390/molecules26051386

**Published:** 2021-03-04

**Authors:** Florent Kerdraon, Gemma Bogard, Benoît Snella, Hervé Drobecq, Muriel Pichavant, Vangelis Agouridas, Oleg Melnyk

**Affiliations:** 1 U1019-UMR 9017—CIIL—Center for Infection and Immunity of Lille, Institut Pasteur de Lille, University of Lille, CNRS, Inserm, CHU Lille, F-59000 Lille, France; florent.kerdraon@ibl.cnrs.fr (F.K.); gemma.bogard@inserm.fr (G.B.); benoit.snella@ibl.cnrs.fr (B.S.); herve.drobecq@ibl.cnrs.fr (H.D.); muriel.pichavant@pasteur-lille.fr (M.P.); 2 Centrale Lille, F-59000 Lille, France

**Keywords:** peptide thioester, alkylselenol, catalysis, chemical protein synthesis

## Abstract

While thiol-based catalysts are widely employed for chemical protein synthesis relying on peptide thioester chemistry, this is less true for selenol-based catalysts whose development is in its infancy. In this study, we compared different selenols derived from the selenocysteamine scaffold for their capacity to promote thiol–thioester exchanges in water at mildly acidic pH and the production of peptide thioesters from *bis*(2-sulfanylethyl)amido (SEA) peptides. The usefulness of a selected selenol compound is illustrated by the total synthesis of a biologically active human chemotactic protein, which plays an important role in innate and adaptive immunity.

## 1. Introduction

**Figure 1:**
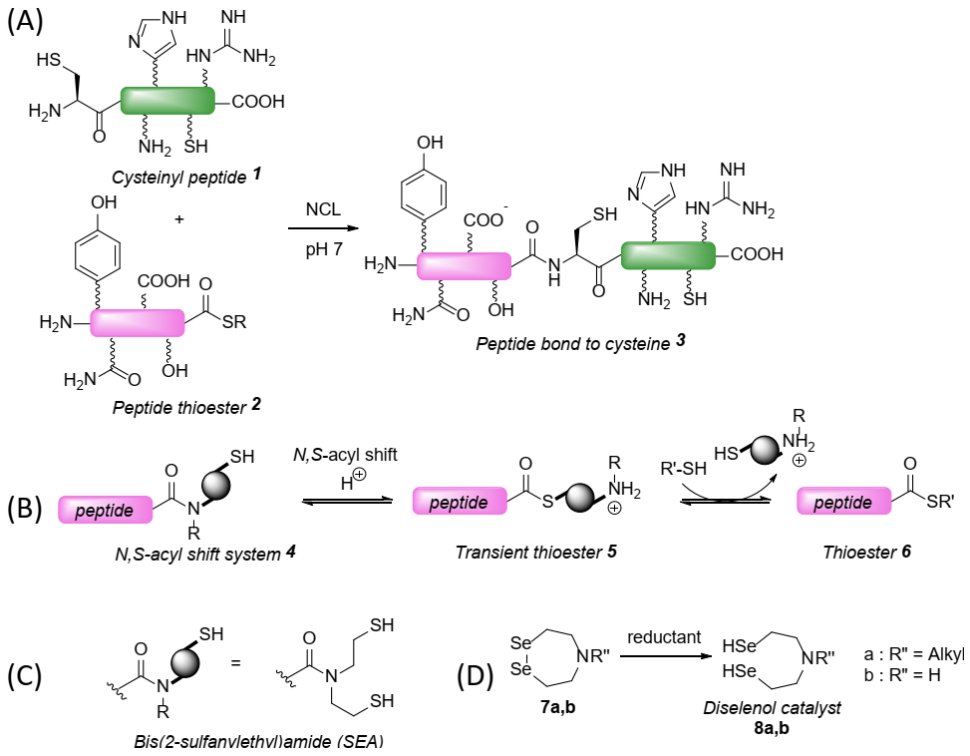
(**A**) Principle of the native chemical ligation (NCL). (**B**) *N,S*-acyl shift amide systems rearrange into thioesters in aqueous solution and can be used for producing stable thioesters by exchange with a thiol. (**C**) The *bis*(2-sulfanylethyl)amide (SEA) system. (**D**) Selenol catalysts reported by Cargoët et al. [47].

Chemical protein synthesis is now established as an alternative mode of production of tailored proteins for use in diverse fields of research, primarily biological and medicinal research [1]. Thioester chemistry is central to the field of chemical protein synthesis by the frequent application of the native chemical ligation (NCL [2,3,4,5,6], Figure 1A) and desulfurization methods [7,8,9,10] to the chemoselective formation of peptide junctions to cysteine or alanine in water using unprotected peptide segments as reactants [11].

Besides the assembly of the protein itself and the acceleration of peptide bond formation [12,13,14,15,16], one limitation of chemical protein synthesis using NCL is the production of the peptide thioester segments [17]. Therefore, facilitating the access to the peptide thioesters using Fmoc-SPPS has been intensively pursued, especially during the last decade, and the progress made in this area explains in part the rapid increase in the number of protein targets assembled since 2010 [1,11]. One corpus of methods that has contributed to the progress of chemical protein synthesis exploits the capacity of *N*-(2-mercaptoethyl)amides, i.e., *N,S*-acyl shift systems [18,19,20,21,22,23,24,25,26,27,28,29,30,31,32,33,34,35,36], to rearrange into thioesters in aqueous media. Several of these systems have been validated by the synthesis of challenging proteins [37,38,39,40,41,42,43,44].

One popular mode of use of *N,S*-acyl shift systems is the production of alkylthioesters by exchanging the *N*-(2-mercaptoethyl)amine unit by a thiol used in excess, i.e., R’SH in Figure 1B. Such exchange reactions are equilibrated, the equilibrium being displaced toward the thioester form at acidic pH probably due to the masking of the departing *N*-(2-mercaptoethyl)amine by protonation [45]. Detailed mechanistic studies performed on the *bis*(2-sulfanylethyl)amido (SEA) system established that in such conditions the thiol–thioester exchanges are rate limiting. This is because thiol–thioester exchanges proceed preferably through thiolate species [46], while the attacking thiol R’SH in the process shown in Figure 1B is mostly in its neutral form at the acidic pH used for the exchange reaction, typically pH 4 [47]. These mechanistic findings led to the conclusion that the production of peptide thioesters from SEA peptides might be facilitated by catalyzing the thiol/thioester exchange step shown in Figure 1B. Indeed, efficient catalysis could be achieved by using diselenol catalyst **8a** obtained by in situ reduction of diselenide **7a** (R” = alkyl group, Figure 1D), which in practice was found to be superior to other classical thiol or selenol additives for promoting the production of peptide thioesters from SEA peptides at pH 4. The design of catalyst **8a** has been inspired by the observation that the simpler diselenol **8b** (R” = H, Figure 1D) was able to catalyze the SEA/thiol exchange reaction, albeit with byproduct formation due to the presence in **8b** of a nucleophilic secondary amine, hence the introduction of a blocking alkyl group on the nitrogen.

**Figure 2:**
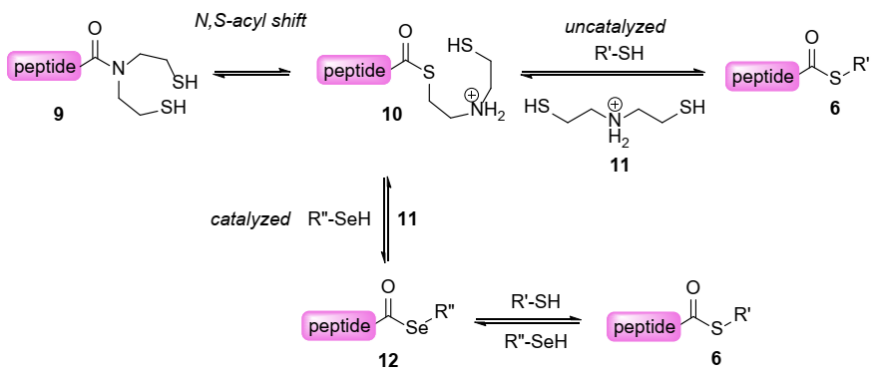
Mechanism of the SEA/thiol exchange catalysis by selenols.

The diverse kinetic data collected so far support that the catalysis of the SEA/thiol exchange reaction by selenols proceeds as shown in Figure 2 [45,47]. According to this mechanism, the SEA peptide **9** undergoes a spontaneous *N,S*-acyl shift to produce the transient SEA thioester **10**. The latter reacts with the selenol catalyst probably through selenoate species to produce selenoester **12**, which then undergoes an exchange reaction with the thiol additive used in excess. The catalytic potency of selenols stems from their low pK_a_ compared to thiols, which ensures that a significant fraction of the selenol is in the form of a nucleophilic selenoate at the working acidic pH for the reaction, and also from the strong acylating properties of selenoesters of type **12** [13,16].

The ease of synthesis, the catalytic potency and the water solubility are certainly key points to consider in the design of catalysts acting on unprotected peptide segments. Diselenol catalysts of type **8a** are efficient and water soluble but their synthesis is complex and low yielding. This latter limitation prompted us to search for novel selenol catalysts whose production would be easier. Evolving catalyst **8a** structure into easier to make selenol catalysts while maintaining similar catalytic potencies is not obvious. This is because the importance of the bis selenol and tertiary amine functionalities in the catalysis by **8a** remains unknown. In addition, the consequences of changing catalyst structure on selenol nucleophilicity and pK_a_ are hard to predict.

**Figure 3:**
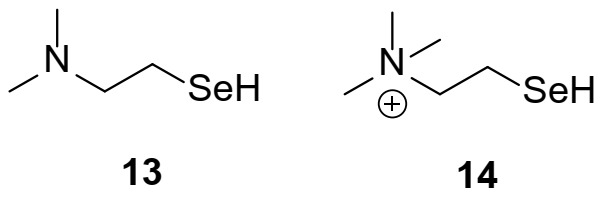
Selenol catalysts studied in this work.

In this work, we evaluated the interest of selenol compounds **13** and **14** for catalyzing the SEA/thiol exchange process (Figure 3). These compounds, which share the 2-(selanylethyl)amino motif with original catalyst **8a**, have been studied in the past as analogs of choline [48,49,50]. They have the advantage of being easily produced in multigram quantities from cheap starting materials. Detailed kinetic studies showed the capacity of selenol **13** to act as a substitute of diselenol **8a** for the synthesis of peptide thioesters. Its usefulness is illustrated by the first chemical synthesis of a biologically active 9 kDa granulysin protein, an antibacterial human protein secreted by cytotoxic cells in response to various infectious agents [51,52,53].

## 2. Results and Discussion

### 2.1. Catalyst Synthesis

**Scheme 1:**
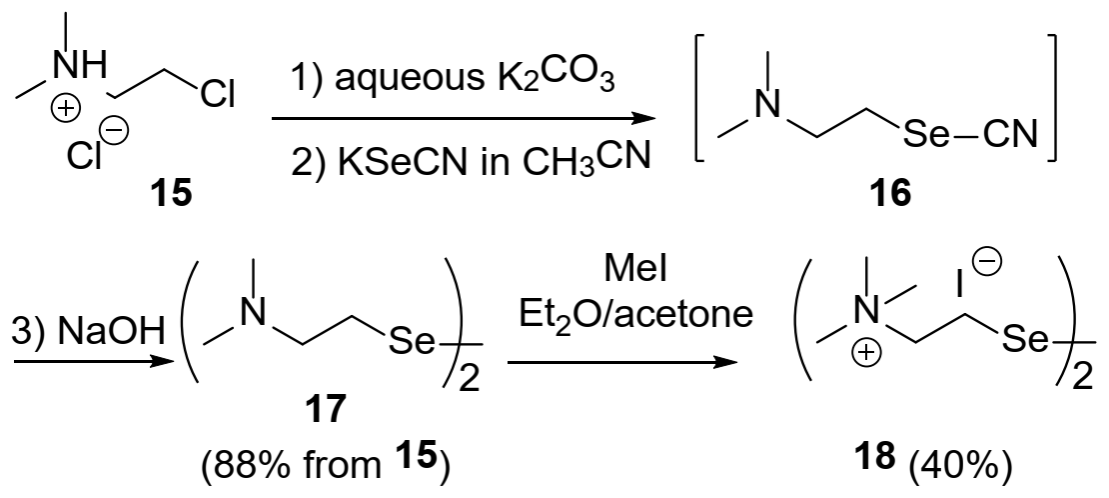
Synthesis of diselenide precatalysts **17** and **18**.

Catalysts **13** and **14** were produced as their corresponding diselenides, i.e., diselenides **17** and **18** respectively, starting from 2-chloro-*N,N*-dimethylethylamine hydrochloride according to known procedures [50,54]. Briefly, 2-chloro-*N,N*-dimethylethylamine was neutralized with potassium carbonate and then reacted with potassium selenocyanate to produce *N*,*N*-dimethyl-2-selenocyanatoethan-1-amine **16**, which upon treatment with sodium hydroxide afforded diselenide **17** (Scheme 1). Diselenide **17** was easily alkylated with methyl iodide to produce diselenide **18**.

### 2.2. Kinetic Studies

**Scheme 2:**
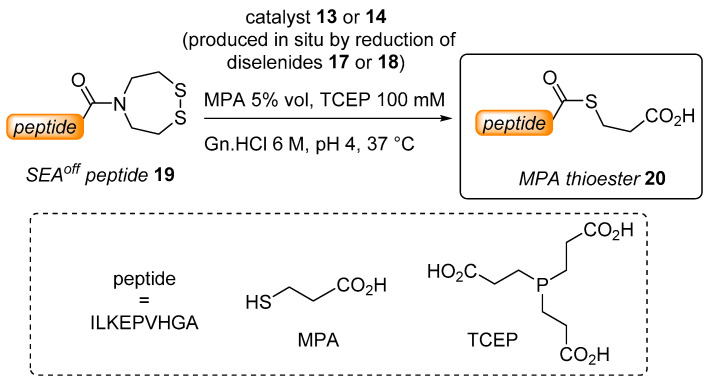
The *bis*(2-sulfanylethyl)amido (SEA)/thiol exchange reaction investigated by kinetic studies.

The SEA/thiol exchange reaction used for studying the potency of the different catalysts is shown in Scheme 2. The water soluble *tris*(2-carboxyethyl)phosphine (TCEP) enables an in situ reduction of the cyclic SEA^off^ disulfide **19** and of the diselenide precatalyst **17** or **18**. The thiol additive used in this reaction is 3-mercaptopropionic acid (MPA), which is a classical thiol used for preparing peptide thioesters.

**Figure 4:**
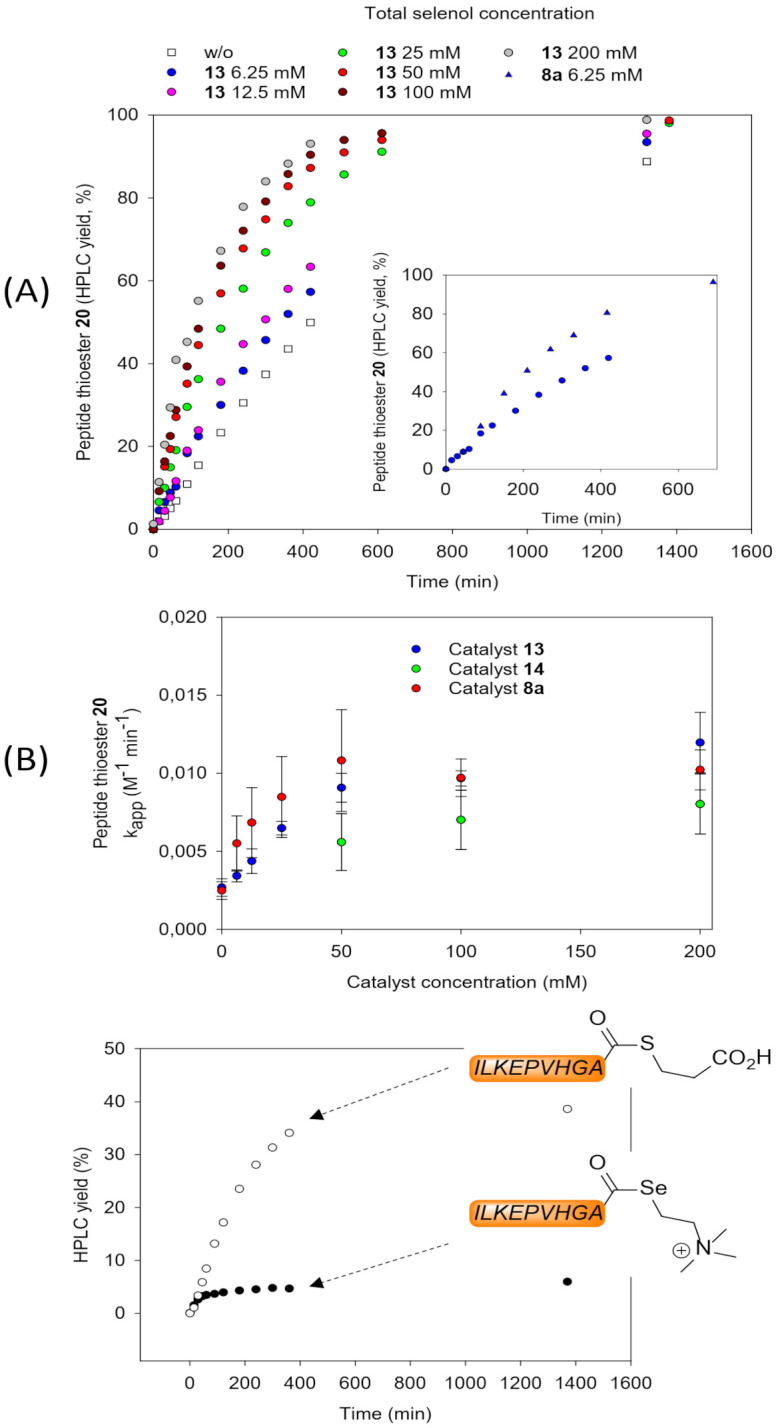
Study of the SEA/thiol exchange reaction catalyzed by selenols **8a** or **13**. Conditions: SEA peptide = 1 mM, 37 °C under inert atmosphere, 6 M Gn∙HCl, MPA 5% *v*/*v*, 100 mM *tris*(2-carboxyethyl)phosphine hydrochloride (TCEP∙HCl), pH 4.0. (**A**) Experimental data as obtained by HPLC monitoring. (**B**) Apparent second-order rate constants obtained by fitting experimental data. The error bars correspond to the standard error of the fit. (**C**) Kinetic profile in the presence of catalyst **14** (200 mM).

The exchange reactions were monitored by HPLC (Figure 4A). The kinetic data were fitted to extract the apparent second order rate constants (Figure 4B). In this figure and in the following paragraphs, the data are expressed as a function of total selenol concentration. As suggested by the experimental data shown in Figure 4B, the rate constants obtained for **13** and **8a,** which are formed by in situ reduction of diselenides **17** and **7a**, plateau at the same value above 50 mM. However, examination of Figure 4B or of Table 1, which gives the half-reaction times for the different experiments, shows that the potencies of catalysts **13** and **8a** are not the same for concentrations below 50 mM. In such a case, the SEA/thiol exchange reaction proceeds faster with catalyst **8a**. The difference is particularly striking by looking at the data obtained at the lowest catalyst concentration tested (compare in the insert of Figure 4A blue circles and triangles for **13** and **8a** respectively used at 6.25 mM concentration). We can conclude at this point that catalyst **8a** can be substituted by selenol **13** with the condition of being used at a minimal concentration of 50 mM, under which conditions the highest rate for the SEA/thiol exchange reaction can be achieved.

The ability to accelerate the SEA/MPA exchange reaction was also assessed according to the same conditions for selenol **14** obtained from in situ reduction of **18**. Whether at a concentration of 200 mM, 100 mM or 50 mM, selenol **14** was found to be significantly less potent than catalysts **8a** and **13** (Table 1). We noticed the formation of a red solid in the reaction mixture after a few hours at the highest concentration tested (200 mM), probably elemental selenium (see Supplementary Information, Figure S17). This observation strongly suggests that catalyst **14** decomposes throughout the course of the reaction. We also noticed an increase of the pH of the reaction mixture, a phenomenon that was not observed with the other selenols. Since previous studies established that the SEA/thioester ratio at equilibrium increases with pH [45,47], the pH drift observed in these experiments might explain in part the diminished conversion observed with catalyst **14**. Taken together, the lower conversion and rates observed with catalyst **14** preclude its use as an interesting alternative to **8a** for the production of peptide thioesters from SEA peptides.

**Figure 5:**
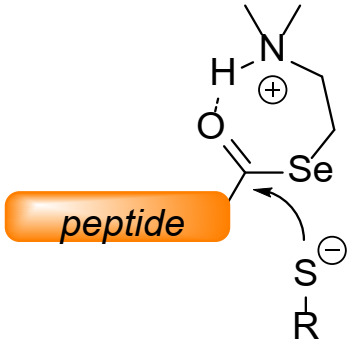
The presence of a protonated amine nearby the peptide carbonyl might promote the attack of thiol nucleophiles.

Another striking observation made during the HPLC-MS monitoring of the exchange process catalyzed by selenol **14** was the formation of the peptide selenoester derived from selenocholine in substantial amounts (Figure 4C). In contrast, the amount of selenoester formed during the SEA/thiol exchange catalyzed by selenols **8a** or **13** was below HPLC-MS detection limits. The persistence of the selenoester derived from **14** in the reaction mixture suggests that the quaternarization of the basic nitrogen significantly alters the reactivity of the carbonyl group nearby. This observation is reminiscent of the work of Mautner and coworkers, who noticed that the hydrolysis of 2-dimethylaminoethyl selenobenzoate proceeded significantly faster at pH 7 than hydrolysis of benzoylselenocholine. The authors proposed that the formation of an intramolecular hydrogen bond between the protonated secondary amine and the selenoester carbonyl oxygen might promote the hydrolysis of the selenoester carbonyl [49]. Such an intramolecular activation of the selenoester group by the protonated amine as illustrated in Figure 5 might contribute to the catalytic potency of selenols **8a** and **13**.

### 2.3. Total Chemical Synthesis of 9 kDa Granulysin

**Figure 6:**
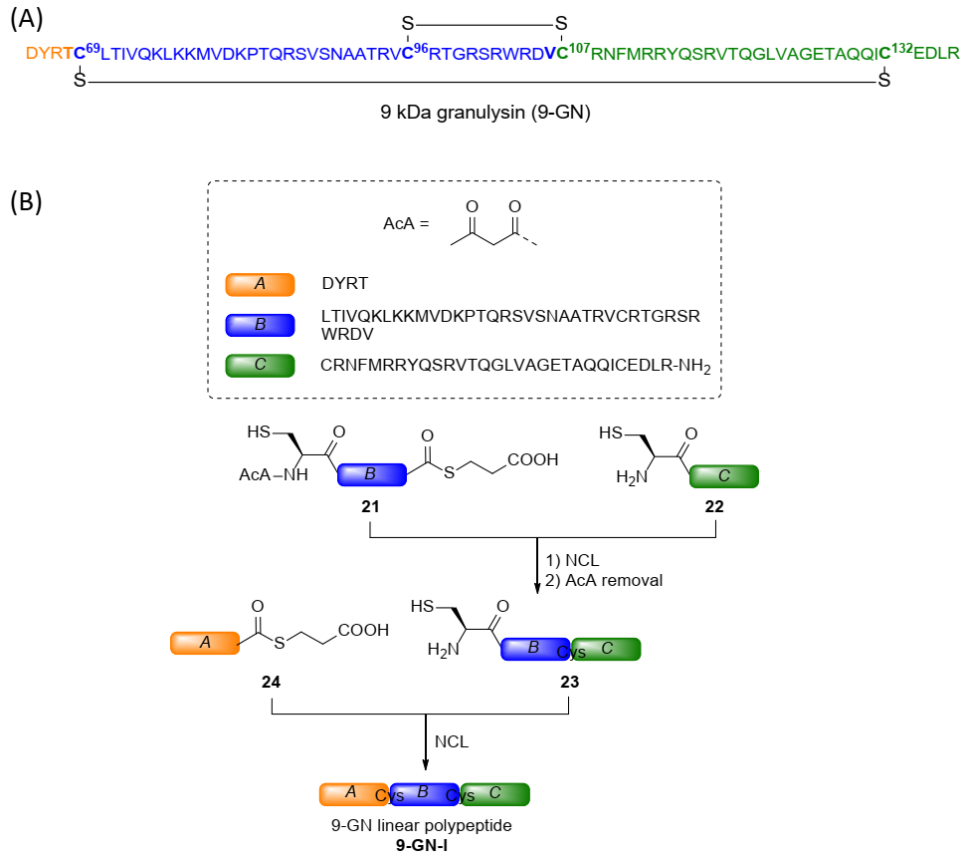
(**A**) Primary structure of 9 kDa granulysin (9-GN) and pattern of disulfide bonds. (**B**) General strategy used for assembling the linear 9-GN polypeptide (**9-GN-l**).

To illustrate the usefulness of the new catalyst **13**, we undertook the total chemical synthesis of 9 kDa granulysin (9-GN, Figure 6A). 9-GN is a human cytotoxic and proinflammatory protein that belongs to the NK-lysin family of proteins [53,55,56]. The protein is stabilized by two disulfide bonds. 9-GN protein is secreted by specialized cells from the immune system in response to infection by various agents such as bacteria, parasites, fungi and viruses. Designing an efficient and modular synthetic approach toward 9-GN can facilitate the development of novel antibacterial proteins of potential therapeutic interest.

The synthetic strategy that was followed for accessing the linear 9-GN polypeptide (**9-GN-l**) is described in Figure 6B. **9-GN-l** polypeptide was assembled from three peptide segments using NCL, which are highlighted by orange (N-terminal), blue (middle) and green (C-terminal) colors in Figure 6. The preceding residue of all the cysteines present in 9-GN sequence are β-branched amino acid residues, meaning that regardless of the AA-Cys junctions chosen for 9-GN assembly, the formation of difficult peptidic junctions will have to be dealt with. The assembly was done in the C-to-N direction and therefore required the protection of the N-terminal cysteine residue of the middle segment to avoid its cyclization or oligomerization during the first ligation step. The acetoacetyl (AcA) group was chosen for this purpose due to its ease of installation and removal [37,38,39,57].

**Figure 7:**
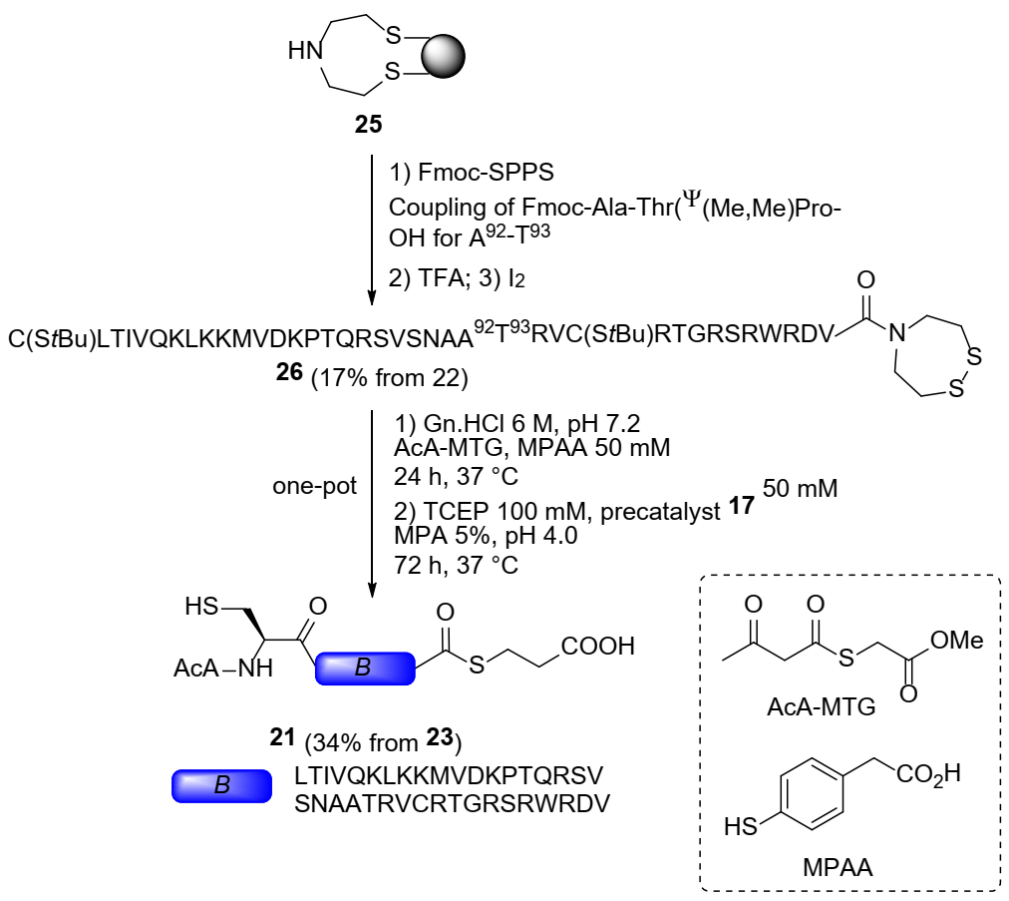
Synthesis of the middle thioester segment **21** by alkyl selenol-catalyzed SEA/MPA exchange.

The preparation of the middle thioester segment **21** in blue in Figure 6B proved to be challenging. First, the classical Fmoc SPPS of the SEA peptide precursor resulted in low yields due to difficult couplings after Arg94. This problem was solved by introducing Ala92 and Thr93 as a pseudoproline dipeptide unit, i.e., Fmoc-Ala-Thr(Ψ(Me,Me)Pro-OH (Figure 7) [58]. The second issue was the sluggish SEA/MPA exchange process enabling the installation of the thioester group, due to the presence of a sterically demanding valine residue at the C-terminus. Catalyst **13** proved to be particularly useful in this case. Interestingly, the protection of the N-terminal cysteine residue using methyl-*S*-(acetoacetyl)-thioglycolate (AcA-MTG) in the presence of 4-mercaptophenylacetic acid (MPAA) and the diselenide-catalyzed SEA/MPA exchange reaction could be performed in one-pot with a pH change in between, thereby saving one intermediate purification step.

**Figure 8:**
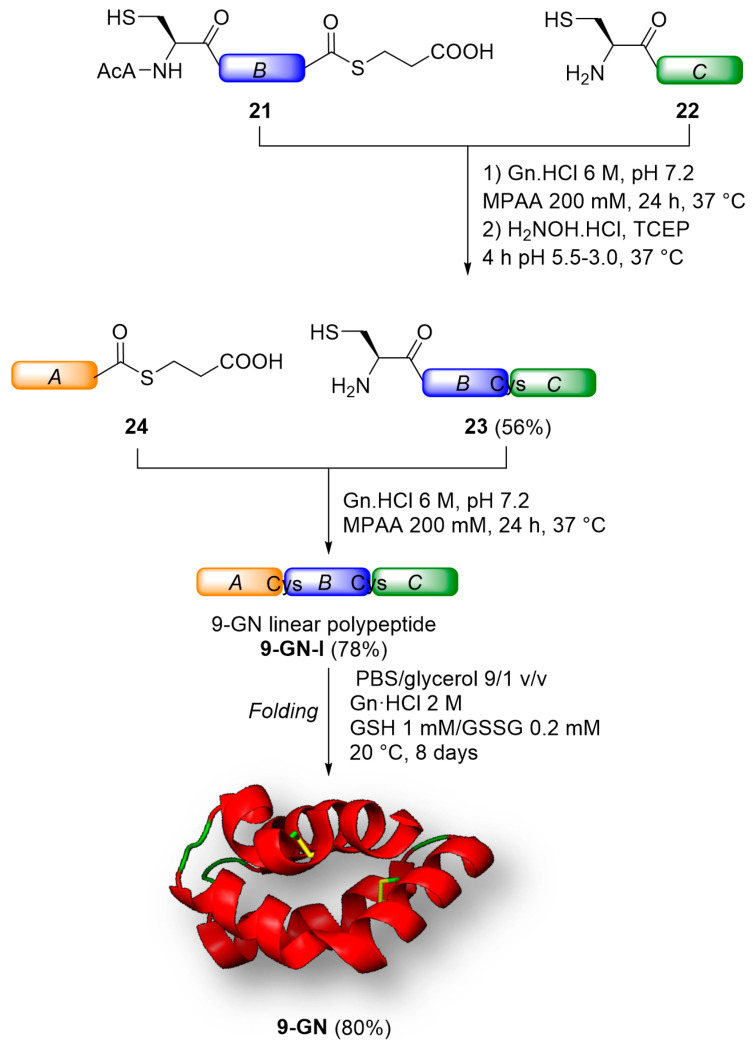
Total synthesis of **9-GN** (the structure of **9-GN** shown in this figure was prepared using Pymol and pdb entry 1L9L [59]).

The successful synthesis of thioester segment **21** set the stage for the assembly of the linear **9-GN-l** polypeptide (Figure 8). The ligation of middle **21** and right **22** segments was followed by the removal of the AcA group in one pot by adding hydroxylamine hydrochloride to the reaction mixture and adjusting the pH to mildly acidic values. The second ligation step with peptide thioester **24** provided the full length **9-GN-l** polypeptide which was purified by HPLC.

**Figure 9:**
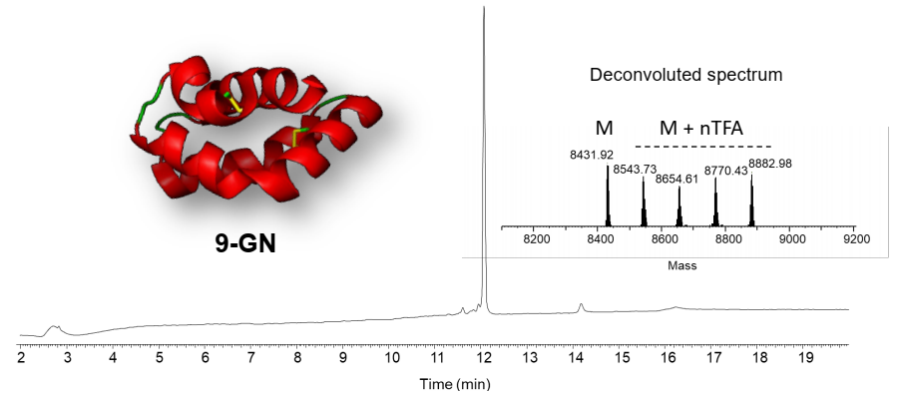
UPLC-MS analysis of synthetic **9-GN**. UV detection at 215 nm. M (average mass) calcd. 8431.79, obs. 8431.9. **9-GN** is a cationic protein that shows several TFA adducts in its deconvoluted MS spectrum. The structure of **9-GN** shown in this figure was prepared using PyMol and pdb entry 1L9L [59].

The folding step was performed at 20 °C in a phosphate buffered saline/glycerol 9/1 mixture in the presence of glutathione (1 mM)/glutathione disulfide (0.2 mM) redox system and guanidine hydrochloride as a solubilizing additive. The use of guanidine hydrochloride during this step was mandatory to enable the solubilisation of the linear **9-GN-l** polypeptide. The formation of the native pattern of disulfide bonds as shown in Figure 6A has been demonstrated by identifying the peptides produced upon trypsin digestion under non-reducing conditions by mass spectrometry (see Supplementary Information). The UPLC-MS analysis of **9-GN** shown in Figure 9 highlights the quality of the protein obtained by the designed synthetic route.

### 2.4. Biological Activity of Synthetic 9-GN

The successful synthesis and folding of 9-GN set the stage for examining its biological activity. One hallmark of 9-GN is its capacity to induce the migration of immune cells to the site of infection. Typically, 9-GN is a chemoattractant for monocytes in the low nanomolar range in a transwell migration assay performed in Boyden micro chambers.

**Figure 10:**
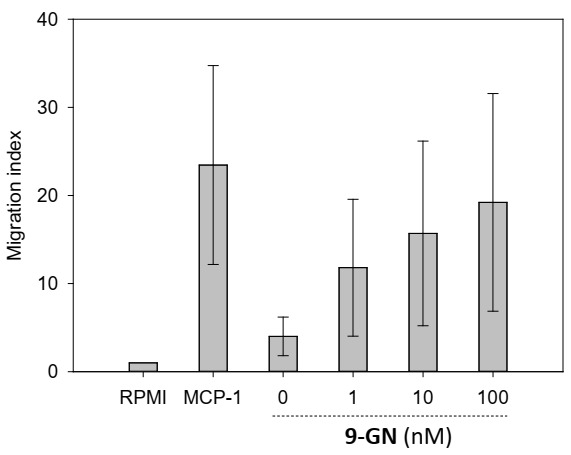
Monocyte migration assay in Boyden micro chambers. The data are expressed relative to the cell migration observed in the Roswell Park Memorial Institute medium (RPMI 1640) supplemented with 0.1% fetal calf serum. Monocyte chemotactic protein-1 (MCP-1, also called CCL2, 23 nM) was used as a positive control. The data for 0 nM of 9-GN were obtained using the protein dialysis buffer (PBS containing 10% by vol of glycerol). The bars correspond to the mean for six donors, from whom monocytes were isolated. The error bar correspond to the standard error of the mean. The data for each donor can be found in the Supplementary Information.

In such an assay, monocytes are placed on the upper layer of a cell permeable membrane inserted in a multi-well plate, which separates the cells from the solution containing the chemoattractant. After incubation (1 h), the cells that have migrated through the membrane are stained and counted. In such an assay, the synthetic 9-GN protein displayed a strong migration index in the 1–100 nM range as expected from literature data (Figure 10), showing the biological functionality of the protein obtained by chemical synthesis [56].

## 3. Materials and Methods

### 3.1. Synthesis of Diselenide Precatalyst 17

To a solution of potassium carbonate (11.5 g, 0.083 mol) in water (30 mL), was added 2-chloroethyl-*N*,*N*-dimethylamine hydrochloride (6.0 g, 0.042 mol). The resulting mixture was stirred at room temperature for 10 min. The resulting crude mixture was extracted 3 times with 30 mL of Et_2_O, dried over MgSO_4_ and concentrated under reduced pressure. 1.0 g of the resulting pale yellow oil (9.3 mmol) was diluted in 90 mL of acetonitrile and potassium selenocyanate (1.47 g, 10.2 mmol) was added portionwise. The resulting solution was stirred at room temperature for 16 h. Et_2_O (100 mL) was added to the reaction mixture which was then filtered through a pad of celite. The filtrate was evaporated in vaccuo and then redissolved in 120 mL of absolute ethanol. Aqueous sodium hydroxide (60 mL, 1 M) was slowly added under stirring and the reaction mixture was stirred 3 h at room temperature. The resulting mixture was concentrated in vacuo to remove the maximum of ethanol and extracted Et_2_O (3 × 30 mL). The organic layers were combined, dried over MgSO_4_ and concentrated under reduced pressure to afford diselenide **17** as a yellow oil (1.24 g, 4.09 mmol, 88%).

^1^H NMR (300 MHz, CDCl_3_) δ (ppm) 3.10–2.96 (m, 4H), 2.68–2.55 (m, 4H), 2.25 (s, 12H) (see Figure S12).

^13^C NMR (75 MHz, CDCl_3_) δ (ppm) 60.32, 45.20, 28.20 (see Figure S13).

MALDI-TOF Matrix 2,5-dihydrobenzoic acid, positive detection mode, calcd. for [M+H]^+^ (exact mass): 305.00, found: 304.92.

### 3.2. Synthesis of 9-GN

Step 1. Synthesis of peptide thioester **21**

To a solution of Gn·HCl (1.1 g) in 0.1 M pH 7.4 phosphate buffer (1.2 mL) was added MPAA (17 mg, 50 mM). SEA^off^ peptide **26** (15 mg, 2.6 μmol, 1.0 equiv) and AcA-MTG (77 µL of a 0.10 M solution in ACN, 7.7 μmol, 3.0 equiv) were then successively dissolved in the MPAA solution (1.3 mL) and the pH of the mixture was adjusted to 7.2 by addition of 6 M NaOH. The mixture was stirred at 37 °C for 8 h. 3.0 additional equivalents of AcA-MTG were added and the mixture was stirred an additional 16 h at 37 °C under nitrogen atmosphere. Completion of the reaction was ascertained by UPLC-MS analysis.

After protection of the N-terminal Cys residue (24 h), the subsequent SEA/MPA exchange reaction was carried out in one pot. TCEP∙HCl (34 mg, 0.10 M), diselenide catalyst **17** (20 mg, 50 mM) and MPA (65 μL, 5% by vol) were added to the previous mixture. The pH was adjusted to 4.0 by addition of 6 M NaOH and the reaction mixture was stirred at 37 °C for 72 h. After completion of the reaction, the mixture was diluted in 10 mL of water containing 0.1% TFA. The crude was purified by HPLC (eluent A = water containing 0.1% TFA, eluent B = acetonitrile in water 4/1 *v*/*v* containing 0.1% TFA, 50 °C, detection at 215 nm, 6 mL min^−1^, 0–37% eluent B in 15 min, then 37–57% eluent B in 60 min, C18XBridge column) to give peptide thioester **21** as a white solid after lyophilization (5.09 mg, 34%).

Peptide **21** was analyzed by UPLC-MS (see Figure S18) and MALDI-TOF mass spectrometry (see Figure S19).

ESI (positive detection mode, see Figure S18): calcd. for [M] (average): 4561.40, found: 4561.79.

MALDI-TOF (positive detection mode, matrix 2,5-dihydrobenzoic acid, see Figure S19): calcd. for [M + H]^+^ (average): 4562.40, found: 4562.87.

Step 2. Synthesis of peptide amide **23**

To a solution of Gn·HCl (0.57 g) in 0.1 M, pH 7.4 phosphate buffer (0.60 mL) was added MPAA (34 mg, 0.20 mmol) and the pH of the mixture was adjusted to 7.2 by addition of 6 M NaOH. Peptide **21** (3.5 mg, 0.61 µmol, 1.0 equiv) and peptide **22** (2.8 mg, 0.67 μmol, 1.1 equiv) were then successively dissolved in the MPAA solution (0.12 mL) and the pH of the mixture was adjusted to 7.2 by addition of 6 M NaOH. The mixture was stirred at 37 °C for 24 h under nitrogen atmosphere. Completion of the reaction was insured by UPLC-MS analysis.

After completion of the NCL ligation (24 h), the AcA protecting group was removed by reaction with NH_2_OH. The reaction mixture was first acidified to pH 3.0 with 6 M HCl and extracted 3 times with 1.0 mL of Et_2_O to remove MPAA. A 0.5 M solution of NH_2_OH·HCl in H_2_O (26 μL, 13 μmol, 20 equiv) was then added and the pH was adjusted to 5.5. After 4 h stirring at 37 °C, pH was adjusted to 3.0 with 0.25 g mL^−1^ aqueous solution of TCEP∙HCl. The crude was further diluted in 4.0 mL of water containing 0.25% TFA and purified by HPLC (eluent A = water containing 0.25% TFA, eluent B = acetonitrile in water 4/1 *v*/*v* containing 0.25% TFA, 50°C, detection at 215 nm, 6 mL min^−1^, 0–40% eluent B in 10 min, then 40–50% eluent B in 45 min, C18XBridge column) to afford peptide **23** as a white solid after lyophilization (3.49 mg, 56%).

Peptide **23** was analyzed by UPLC-MS (see Figure S20) and MALDI-TOF mass spectrometry (see Figure S21).

ESI (positive detection mode, see Figure S20): calcd. for [M] (average): 7899.22, found: 7899.94.

MALDI-TOF (positive detection mode, matrix 2,5-dihydrobenzoic acid, see Figure S21): calcd. for [M+H]^+^ (average): 7900.22, found: 7901.49.

Step 3. Synthesis of **9-GN-l**

To a solution of Gn·HCl (0.57 g) in 0.1 M pH 7.4 phosphate buffer (0.60 mL) was added MPAA (34 mg, 0.20 mmol) and the pH of the mixture was adjusted to 7.7 by addition of 6 M NaOH. Peptide **23** (3.5 mg, 0.36 μmol, 1.0 equiv) and peptide thioester **24** (0.38 mg, 0.36 μmol, 1.0 equiv) were then successively dissolved in the MPAA solution (91 µL) The mixture was stirred at 37 °C for 24 h under nitrogen atmosphere. Completion of the reaction was insured by UPLC-MS analysis.

After completion of the NCL ligation (24 h), TCEP∙HCl (5.2 mg, 18 µmol) was added to the reaction medium to reduce any mixed disulfide formed during ligation. The resulting mixture was diluted in 3.0 mL of water containing 0.1% TFA. The crude was purified by HPLC (eluent A = water containing 0.1% TFA, eluent B=acetonitrile in water 4/1 *v*/*v* containing 0.1% TFA, 50 °C, detection at 215 nm, 6 mL min^−1^, 0–38% eluent B in 10 min, then 38–58% eluent B in 60 min, C18XBridge column) to give **9-GN-l** as a white solid after lyophilization (2.92 mg, 78%).

Peptide **9-GN-l** was analyzed by UPLC-MS (see Figure S22) and MALDI-TOF mass spectrometry (see Figure S23).

ESI (positive detection mode, see Figure S22): calcd. for [M] (average): 8435.79, found: 8435.45.

MALDI-TOF (positive detection mode, matrix 2,5-dihydrobenzoic acid, see Figure S23): calcd. for [M + H]^+^ (average): 8436.79, found: 8436.65.

### 3.3. Folding

The linear polypeptide **9-GN-l** (0.558 mg, 0.054 µmol) was dissolved in PBS containing 10% by vol of glycerol, 2 M of Gn·HCl, 1 mM reduced glutathione, 0.2 mM oxidized glutathione (1 mL/mg final peptide concentration). The reaction mixture was let 1 h at 0 °C, then 8 days at 20 °C.

The protein was dialyzed twice (respectively 2 h and 24 h) at 20 °C against 200 mL PBS containing 10% in vol of glycerol. 0.71 mL of the dialyzed solution were recovered and the concentration of the folded protein was determined using UV quantification at 280 nm (60.7 µM, 0.043 µmol, 80%)

Folded protein **9-GN** was analyzed by UPLC-MS (see Figure S24).

Experimental determination of the disulfide bridge pattern was achieved by trypsin digestion of **9-GN** and identification of the resulting fragments by mass spectrometry using non-reducing conditions. Fragments obtained by digestion with trypsin permitted the direct assignment of Cys69-Cys132 and Cys96-Cys107 disulfide bonds (see Figure S26 in the Supplementary Information).

### 3.4. Chimiotaxis Assay

Blood monocytes were purified from six donors by positive selection over a MACS column using anti-CD14-conjugated microbeads (Miltenyi Biotec) as previously described [60].

Monocyte migration (5 × 10^4^ cells/well in 50 μL of RPMI 1640 medium with 0.1% FCS) was performed in a 48-well Boyden microchamber (Neuroprobe) through a standard 5-μm pore filter (Neuroprobe), at 37 °C for 1 h. Migration was evaluated in response to synthetic **9-GN** (1 to 100 nM in RPMI 1640 medium containing 0.1% FCS), CCL2 used as positive control (200 ng/mL, 23.1 nM, R&D Systems), and medium alone used as negative control. The filter was stained using Diff–Quik reagent. Migrated cells were counted on the lower side of the filter in three randomly selected high power fields (magnification × 100). Each assay was performed in triplicate. Results are expressed as the difference between mean numbers of cells per high power fields minus the negative control (medium alone).

## 4. Conclusions

We conducted detailed kinetic studies of the catalysis of the *bis*(2-sulfanylethyl)amido (SEA)/thiol exchange process by selenols derived from selenocysteamine. These data provide important clues regarding the role played by the basic amino group of the selenocysteamine scaffold in catalysis and how to evolve the catalyst to achieve useful catalytic potencies while minimizing synthetic cost. Catalysis by *bis*(*N*,*N*-dimethylaminoethyl)diselenide proved particularly useful and involves presumably an intramolecular general acid catalysis by the proton of the dimethylammonium group. *Bis*(*N*,*N*-dimethylaminoethyl)diselenide can be easily produced in multigram scale from cheap starting materials. Its usefulness is illustrated by the chemical synthesis of a biologically actice 9 kDa granulysin.

## Figures and Tables

**Table 1:** Catalysis of *bis*(2-sulfanylethyl)amido (SEA)–thiol exchange using selenols **8a**, **13** or **14** ^a,b^.

Entry	Total Selenol Concentration 8a, 13, 14 (mM)	Catalyst t_1/2_ (h)		
		8a ^c^	13	14 ^d^
1	200	1.97	1.68	2.51
2	100	2.03	2.08	2.87
3	50	1.95	2.22	3.60
4	25	2.35	3.10	nd
5	12.5	3.00	4.60	nd
6	6.25	3.35	5.87	nd

^a^ Conditions: SEA peptide = 1 mM, 37 °C under inert atmosphere, 6 M Gn∙HCl, MPA 5% *v*/*v*, 100 mM *tris*(2-carboxyethyl)phosphine hydrochloride (TCEP∙HCl), pH 4.0. Selenol catalysts were added in the form of the corresponding diselenides. ^b^ The half-reaction time (t ½) for the uncatalyzed reaction is 7.28 h [47]. ^c^ Data from ref [47]. ^d^ Measured pH at equilibrium: pH 4.45 at 200 mM **14**, pH 4.19 at 100 mM **14**, pH 4.14 at 50 mM **14**.

## Data Availability

Not applicable.

## Supplementary Materials

The detailed experimental procedures and characterization data for all compounds are provided in a separate PDF file, Figure S1: General approach for the preparation of peptide amides, Figure S2: Synthesis of SEAoff peptides and MPA peptide thioesters, Figure S3: UPLC-MS analysis of CRNFMRRYQSRVTQGLVAGETAQQICEDLR-NH2 peptide 22. LC trace: eluent A 0.10% TFA in water, eluent B 0.10% TFA in CH3CN. C18 column, gradient 0–70% B in 20 min, 50 °C, 0.4 mL min-1, UV detection at 215 nm. MS trace: m/z 1765.33 ([M+2H]2+), 1177.33 ([M+3H]3+), 883.33 ([M+4H]4+), 706.75 ([M+5H]5+); calcd. for [M] (average mass): 3529.04, found: 3529.04, Figure S4: MALDI-TOF analysis of peptide 22. Matrix 2,5-dihydrobenzoic acid, positive detection mode, calcd. for [M+H]+ (average): 3530.04, found: 3529.8, Figure S5: UPLC-MS analysis of C(S*t*Bu)LTIVQKLKKMVDKPTQRSVSNAATRVC(S*t*Bu)-RTGRSRWRDV-SEAoff peptide 26. LC trace: eluent A 0.10% TFA in water, eluent B 0.10% TFA in CH3CN. C18 column, gradient 0–70% B in 20 min, 50 °C, 0.4 mL min-1, UV detection at 215 nm. MS trace: m/z 1636.25 ([M+2MTFA+3H]3+), 1598.92 ([M+MTFA+3H]3+), 1199.42 ([M+MTFA+4H]4+), 1171.75 ([M+4H]4+), 959.92 ([M+MTFA+5H]5+), 937.58 ([M+5H]5+), 781.67 ([M+6H]6+), 670.67 ([M+7H]7+); calcd. for [M] (average mass): 4682.77, found: 4682.95, Figure S6: MALDI-TOF analysis of peptide 26. Matrix 2,5-dihydrobenzoic acid, positive detection mode, calcd. for [M+H]+ (average): 4683.77, found: 4684.22, Figure S7: UPLC-MS analysis of DYRT-SEAoff peptide. LC trace: eluent A 0.10% TFA in water, eluent B 0.10% TFA in CH3CN. C18 column, gradient 0–70% B in 20 min, 50 °C, 0.4 mL min-1, UV detection at 215 nm. MS trace: m/z 671.42 ([M+H]+); calcd. for [M+H]+ (monoisotopic): 671.26, found: 671.42, Figure S8: MALDI-TOF analysis of DYRT-SEAoff peptide. Matrix 2,5-dihydrobenzoic acid, positive detection mode, calcd. for [M+H]+ (monoisotopic): 671.26, found: 671.15, Figure S9: UPLC-MS analysis of DYRT-MPA peptide 24. LC trace: eluent A 0.10% TFA in water, eluent B 0.10% TFA in CH3CN. C18 column, gradient 0–70% B in 20 min, 50 °C, 0.4 mL min-1, UV detection at 215 nm. MS trace: m/z 1283.17 ([2M+H]+), 642.42 ([M+H]+); calcd. for [M+H]+ (monoisotopic): 642.25, found: 642.42, Figure S10: MALDI-TOF analysis of peptide 24. Matrix 2,5-dihydrobenzoic acid, positive detection mode, calcd. for [M+H]+ (monoisotopic): 642.25, found: 642.18, Figure S11: Synthesis scheme for the preparation of diselenide precatalyst 17, Figure S12: 1H NMR (300 MHz) spectrum of compound 17 (CDCl3, 293 K), Figure S13: 13C APT NMR (75 MHz) spectrum of compound 17 (CDCl3, 293 K), Figure S14: 1H NMR (300 MHz) spectrum of compound 18 (D2O, 293 K), Table S1: Kinetic data extracted from curve fitting.^a^, Table S2: Fitting of MPA thioester formation in the presence of catalysts.^a^, Figure S16: Fitting of SEA/MPA exchange in the presence of catalyst 13 (200 mM). Data were fitted according to equation (1), Figure S17: Red selenium precipitate formed during catalysis of SEA/MPA exchange in the presence of catalyst 14 (200 mM), Figure S18: UPLC-MS analysis of peptide 21. LC trace: eluent A 0.10% TFA in water, eluent B 0.10% TFA in CH3CN. C18 column, gradient 0–70% B in 20 min, 50 °C, 0.4 mL min-1, UV detection at 215 nm. MS trace: m/z 1558.25 ([M+MTFA+3H]3+), 1521.42 ([M+3H]3+), 1169.00 ([M+MTFA+4H]4+), 1141.58 ([M+4H]4+), 913.58 ([M+5H]5+), 761.58 ([M+6H]6+), 652.83 ([M+7H]7+); calcd. for [M] (average mass): 4561.40, found: 4561.79, Figure S19: MALDI-TOF analysis of peptide 21. Matrix 2,5-dihydrobenzoic acid, positive detection mode, calcd. for [M+H]+ (average): 4562.40, found: 4562.87, Figure S20: UPLC-MS analysis of peptide 23. LC trace: eluent A 0.10% TFA in water, eluent B 0.10% TFA in CH3CN. C18 column, gradient 0–70% B in 20 min, 50 °C, 0.4 mL min-1, UV detection at 215 nm. MS trace: calcd. for [M] (average mass): 7899.22, found: 7899.94 (8012.58 [M+MTFA], 8124.97 [M+2MTFA], 8238.28 [M+3MTFA]), Figure S21: MALDI-TOF analysis of peptide 23. Matrix 2,5-dihydrobenzoic acid, positive detection mode, calcd. for [M+H]+ (average): 7900.22, found: 7901.49, Figure S22: UPLC-MS analysis of peptide 9-GN-l. LC trace: eluent A 0.10% TFA in water, eluent B 0.10% TFA in CH3CN. C18 column, gradient 0–70% B in 20 min, 50 °C, 0.4 mL min-1, UV detection at 215 nm. MS trace: calcd. for [M] (average mass): 8435.79, found: 8435.45 (8547.50 [M+MTFA], 8659.48 [M+2MTFA]), Figure S23: MALDI-TOF analysis of peptide 9-GN-l. Matrix 2,5-dihydrobenzoic acid, positive detection mode, calcd. for [M+H]+ (average): 8436.79, found: 8436.65, Figure S24: UPLC-MS analysis of 9-GN. LC trace: eluent A 0.10% TFA in water, eluent B 0.10% TFA in CH3CN. C18 column, gradient 0–70% B in 20 min, 50 °C, 0.4 mL min-1, UV detection at 215 nm. MS trace: calcd. for [M] (average mass): 8431.79, found: 8431.92 (8543.73 [M+MTFA], 8654.61 [M+2MTFA], 8770.43 [M+3MTFA], 8882.98 [M+4MTFA]), Figure S25: Disulfide bridge pattern of 9-GN, Figure S26: MALDI-TOF analysis of the trypsic digest of 9-GN. Matrix 2,5-dihydroxybenzoic acid (DHB), positive detection mode.VC96R linked to DVC107R by disulfide bridge, calcd. (monoisotopic) [M+H+] 866.40, found 866.39; VC96R linked to WRDVC107R by disulfide bridge, calcd. (monoisotopic) [M+H+] 1208.58, found 1208.57; TC69LTIVQK linked to VTQGLVAGETAQQIC132EDLR by disulfide bridge, calcd. (monoisotopic) [M+H+] 2932.52, found 2932.49; DYRTC69LTIVQK linked to VTQGLVAGETAQQIC132EDLR by disulfide bridge, calcd. (monoisotopic) [M+H+] 3366.71, found 3366.73, Table S3: Results of the Boyden micro chamber assay.
