# Supplementary material for: Insights into the Mechanism and Catalysis of Peptide Thioester Synthesis by Alkylselenols Provide a New Tool for Chemical Protein Synthesis
Source: Molecules. 2021 Mar 4;26(5):1386. doi: 10.3390/molecules26051386 (PMC7961367; doi:10.3390/molecules26051386)
Supplement: Supplementary file 1 [file molecules-26-01386-s001.pdf]

Insights into the mechanism and catalysis of peptide thioester synthesis by alkylselenols provide a new tool for chemical protein synthesis

Florent Kerdraon,[a] Gemma Bogard,[a] Benoît Snella,[a] Hervé Drobecq,[a] Muriel Pichavant,[a] Vangelis Agouridas,[a,b]* Oleg Melnyk[a]*

[a] Univ. Lille, CNRS, Inserm, CHU Lille, Institut Pasteur de Lille, U1019 - UMR 9017 - CIIL - Center for Infection and Immunity of Lille, F-59000 Lille, France

[b] Centrale Lille, F-59000 Lille, France

Corresponding authors

oleg.melnyk@ibl.cnrs.fr

vangelis.agouridas@ibl.cnrs.fr

1. Table of content

1. Table of content **2**

2. General methods **3**

3. Peptide synthesis **4**

3.1. General procedures *4*

3.2. Synthesis and characterization of peptide amides *6*

3.3. Synthesis and characterization of SEA^{off} peptides *8*

3.4. Synthesis and characterization of MPA thioester peptides *12*

4. Synthesis of diselenide precatalysts **15**

4.1. Diselenide precatalyst 17 .... *15*

4.2. Diselenide precatalyst 18 .... *17*

1. Kinetic studies and modelisation **19**

5.1. Generalities *19*

5.2. Rate constants of uncatalyzed and catalyzed SEA peptide / MPA exchange reactions *19*

5.3 Analytical approach *22*

2. Synthesis of linear 9 kDa granulysin polypeptide 9-GN-l **24**

3. Folding of 9-GN-l and characterization of 9-GN **28**

4. Boyden-type microchamber chemotaxis assay **32**

2. General methods

Reagents and solvents

1-[*bis*(dimethylamino)methylene]-1*H*-1,2,3-triazolo[4,5-*b*]pyridinium 3-oxid hexafluorophosphate (HATU), 2-(1*H*-Benzotriazol-1-yl)-1,1,3,3-tetramethyluronium hexafluorophosphate (HBTU), O-(1*H*-6-chlorobenzotriazole-1-yl)-1,1,3,3-tetramethyluronium hexafluorophosphate (HCTU), and *N*-Fmoc protected amino acids were obtained from Iris Biotech GmbH. Side-chain protecting groups used for the amino acids were Fmoc-Arg(Pbf)-OH, Fmoc-Asn(Trt)-OH, Fmoc-Asp(O*t*Bu)-OH, Fmoc-Gln(Trt)-OH, Fmoc-Glu(O*t*Bu)-OH, Fmoc-His(Trt)-OH, Fmoc-Lys(Boc)-OH, Fmoc-Ser(*t*Bu)-OH, Fmoc-Thr(*t*Bu)-OH, Fmoc-Trp(Boc)-OH, Fmoc-Tyr(*t*Bu)-OH, Fmoc-Cys(S*t*Bu)-OH or Fmoc-Cys(Trt)-OH. Fmoc-Ala-Thr(Ψ(Me,Me)Pro-OH was obtained from Bachem.

The synthesis of *bis*(2-sulfanylethyl)aminotrityl polystyrene (SEA PS) solid support was carried out as described elsewhere.[1,2] Synthesis of bis(2-sulfanylethyl)aminotrityl ChemMatrix® (SEA ChemMatrix®) resin was carried out as described elsewhere.[3,4] 4-Mercaptophenylacetic acid (97%, MPAA), 3-mercaptopropionic acid (MPA), *tris*(2-carboxyethyl)phosphine hydrochloride (≥ 98%), TCEP), triisopropylsilane (TIS), dimethyl sulfide (DMS), guanidine hydrochloride (Gn·HCl, ≥ 99%), sodium phosphate dibasic dihydrate (≥ 99%), hydrochloric acid (reagent grade, 37% w/v) and sodium hydroxide (pellets, 97%) were purchased from Sigma-Aldrich. All other reagents were purchased from Acros Organics or Merck and were of the purest grade available.

Peptide synthesis grade *N*,*N*-dimethylformamide (DMF), dichloromethane (CH_2Cl_2), diethylether (Et_2O), acetonitrile (CH_3CN), heptane, LC–MS-grade acetonitrile (CH_3CN, 0.1% TFA), LC–MS-grade water (H_2O, 0.1% TFA), *N*,*N*-diisopropylethylamine (DIEA), acetic anhydride (Ac_2O) were purchased from Biosolve and Fisher-Chemical. Trifluoroacetic acid (TFA) was obtained from Biosolve. Formic acid (FA) reagent grade was obtained from Sigma Aldrich. Water was purified with a Milli-Q Ultra Pure Water Purification System.

---

[1] Ollivier, N.; Dheur, J.; Mhidia, R.; Blanpain, A.; Melnyk, O. *Bis*(2-Sulfanylethyl)Amino Native Peptide Ligation. *Org. Lett.* **2010**, *12*, 5238-5241.

[2] Ollivier, N.; Raibaut, L.; Blanpain, A.; Desmet, R.; Dheur, J.; Mhidia, R.; Boll, E.; Drobecq, H.; Pira, S. L.; Melnyk, O. Tidbits for the Synthesis of *Bis*(2-Sulfanylethyl)Amido (SEA) Polystyrene Resin, SEA Peptides and Peptide Thioesters. *J. Pept. Sci.* **2014**, *20*, 92–97.

[3] Boll, E.; Drobecq, H.; Ollivier, N.; Blanpain, A.; Raibaut, L.; Desmet, R. *et al.* One-pot chemical synthesis of small ubiquitin-like modifier (SUMO) protein-peptide conjugates using *bis*(2-sulfanylethyl)amido peptide latent thioester surrogates *Nat. Protoc.* **2015**, *10*, 269-292.

[4] Boll, E.; Drobecq, H.; Ollivier, N.; Raibaut, L.; Desmet, R.; Vicogne, J.; Melnyk, O. A novel PEG-based solid support enables the synthesis of > 50 amino-acid peptide thioesters and the total synthesis of a functional SUMO-1 peptide conjugate *Chem Sci* **2014**, *5*, 2017-2022.

Analyses

Products were characterized by analytical UPLC-MS using a System Ultimate 3000 UPLC (Thermofisher) equipped with a diode array detector, a charged aerosol detector (CAD) and a mass spectrometer (Ion trap LCQfleet). The column eluate was monitored by UV at 215 nm and CAD. The peptide masses were measured by on-line UPLC-MS (LCQ Fleet Ion Trap Mass Spectrometer, ThermoFisherScientific). Unless otherwise specified, heat temperature is set at 350 °C, spray voltage at 2.8 kV, capillary temperature at 350 °C, capillary voltage at 10 V, tube lens voltage at 75 V.

MALDI-TOF mass spectra were recorded with a Bruker Autoflex Speed using alpha cyano 4-hydroxycinnaminic acid, sinapinic acid or 2,5-dihydroxybenzoic acid as matrix. The observed m/z corresponded to the monoisotopic ions, unless otherwise stated.

Purifications

Preparative reverse phase HPLC of crude peptides were performed with a preparative HPLC Waters system using the appropriate linear gradient of increasing concentration of eluent B in eluent A (flow rate of 6 mL min^{-1}, detection at 215 nm). Selected fractions were then combined and lyophilized.

3. Peptide synthesis

3.1. General procedures

Peptide amides were synthesized on a NovaSyn TGR solid support (0.25 mmol/g) using standard Fmoc chemistry and an automated peptide synthesizer (Figure S 1)

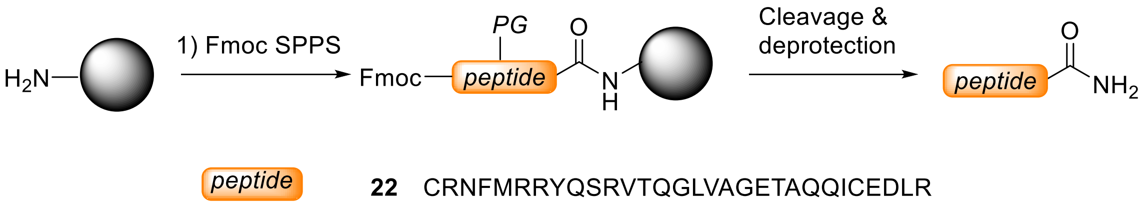

Figure S 1. General approach for the preparation of peptide amides.

SEA^{off} peptides were synthesized on bis(2-sulfanylethyl)aminotrityl polystyrene solid support (SEA PS) (0.16 mmol/g) or on SEA ChemMatrix® solid support using standard Fmoc chemistry and an automated peptide synthesizer (Figure S 2). The SEA^{on} peptide obtained after acidic cleavage was converted into the corresponding SEA^{off} peptide by oxidation with iodine. The same intermediate provided the MPA thioester *via* a SEA/MPA exchange reaction. Detailed procedures to prepare these peptides are available in previous publications.[3,5,6] Note that the first amino acid was directly linked to the solid support.

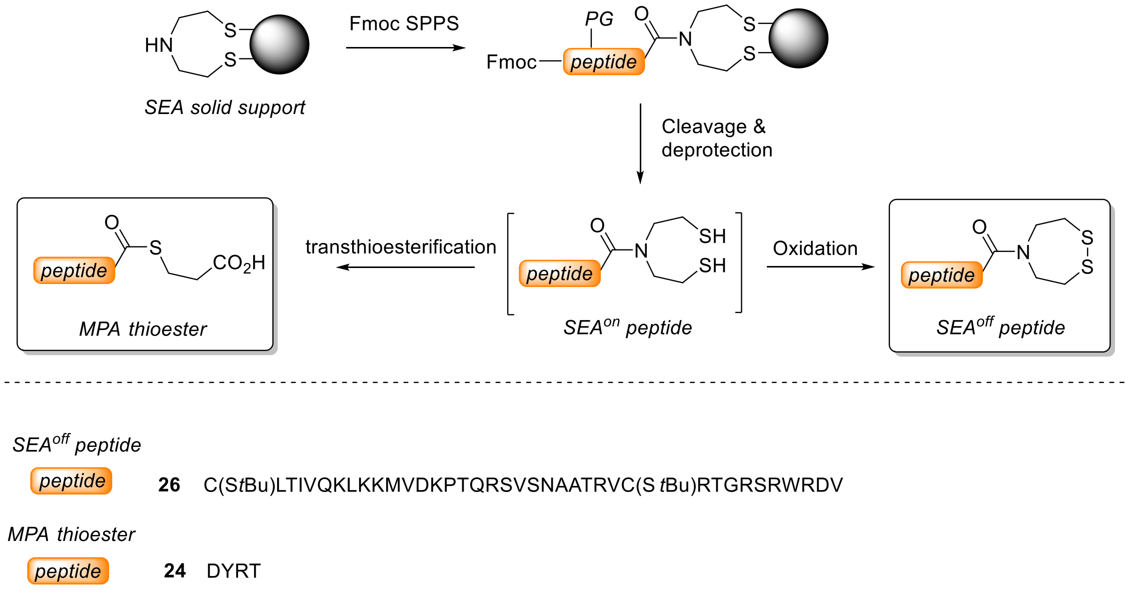

Figure S 2. Synthesis of SEA^{off} peptides and MPA peptide thioesters.

Coupling of the first amino acid on SEA PS and SEA ChemMatrix® beads

The first amino acid (10 equiv) was coupled to SEA PS solid support (1.0 equiv) using HATU (10 equiv)/DIEA (20 equiv) activation in DMF. The amino acid was preactivated for 2 min and then added to the beads swelled in the minimal volume of DMF. The beads were agitated at RT for 1.5 h, then washed with DMF (3 × 2 min) and drained. The absence of unreacted secondary amino groups was checked using the chloranyl colorimetric assay. A capping step was then performed using Ac_2O/DIEA/DMF 10/5/85 v/v/v (2 × 5 min) and the beads were washed with DMF (3 × 2 min).

Automated peptide elongation

Peptide elongation was performed using standard Fmoc chemistry on an automated peptide synthesizer without microwaves. Couplings were performed using a 4-fold molar excess of each Fmoc L-amino acid, a 3.6-fold molar excess of HBTU and an 8-fold molar excess of DIEA. A

[5] Ollivier, N.; Raibaut, L.; Blanpain, A.; Desmet, R.; Dheur, J.; Mhidia, R. *et al.* Tidbits for the synthesis of *bis*(2-sulfanylethyl)amido (SEA) polystyrene resin, SEA peptides and peptide thioesters. *J. Pept. Sci.* **2014**, *20*, 92–97 .
[6] Ollivier, N.; Dheur, J.; Mhidia, R.; Blanpain, A; Melnyk, O. *Bis*(2-sulfanylethyl)amino native peptide ligation. *Org. Lett.* **2010**, *12*, 5238-5241.

capping step was performed with Ac_2O/DIEA/DMF 10/5/85 v/v/v before Fmoc removal using piperidine/DMF 80/20 v/v.

Final peptide deprotection and cleavage

At the end of the synthesis, the beads were washed with DCM (3 × 2 min) and diethyl ether (3 × 2 min) and dried *in vacuo*. The crude peptide was cleaved from the solid support using TFA cleavage and deprotection cocktails, precipitated by addition of cold diethyl ether/*n*-heptane 1/1 v/v (20 mL per mL of TFA cocktail) and recovered by centrifugation.

Oxidation of the SEA group (SEA^{on} → SEA^{off})

The SEA^{on} peptide recovered by centrifugation was solubilized in deionized water, lyophilized and dissolved in AcOH/water 1/4 v/v. A solution of I_2 in DMSO (≈ 100 mg mL^{-1}) was added dropwise until complete oxidation of the SEA group (persistence of the yellow color of iodine in the reaction mixture). After 30 s of stirring, a solution of DTT in AcOH/water 1/4 v/v (≈ 100 mg mL^{-1}) was added to consume the excess of I_2. Then the peptide was immediately purified by HPLC.

Transthioesterification (SEA^{off} → MPA)

The SEA^{off} peptide recovered after purification was converted into the corresponding MPA thioester by reaction with MPA (5% in volume) at pH 4.0. A detailed procedure is given below for the synthesis of MPA thioesters.

3.2.Synthesis and characterization of peptide amides

CRNFMRRYQSRVTQGLVAGETAQQICEDLR-NH_2 peptide **22** was synthesized on 0.10 mmol scale as described in the general procedure and was cleaved from the solid support using TFA/TIS/H_2O/EDT 94.5/5/2.5/2.5 v/v/v/v (10 mL). The title peptide was recovered by precipitation from Et_2O/n-heptane 1/1 v/v, solubilized in water and lyophilized. Purification of the crude by semi-preparative HPLC (eluent A 0.10% TFA in water, eluent B 0.10% TFA in CH_3CN/water 4/1 v/v, 6 mL min^{-1}, 0-28% eluent B in 10 min and then 28-38% eluent B in 40 min, C18 XBridge 5 µm (19 × 150 mm) column, detection at 215 nm, 50 °C, provided peptide **22** as a white solid after lyophilization (78.8 mg, 18%).

Characterization of CRNFMRRYQSRVTQGLVAGETAQQICEDLR-NH_2 peptide **22***:*

Peptide **22** was analyzed by UPLC-MS (Figure S 3) and MALDI-TOF mass spectrometry (Figure S 4).

ESI (positive detection mode, see Figure S 3): calcd. for **[**M] (average mass): 3529.04, found: 3529.04.

MALDI-TOF (positive detection mode, matrix 2,5-dihydrobenzoic acid, Figure S 4): calcd. for $[M+H]^+$ (average): 3530.04, found: 3529.8.

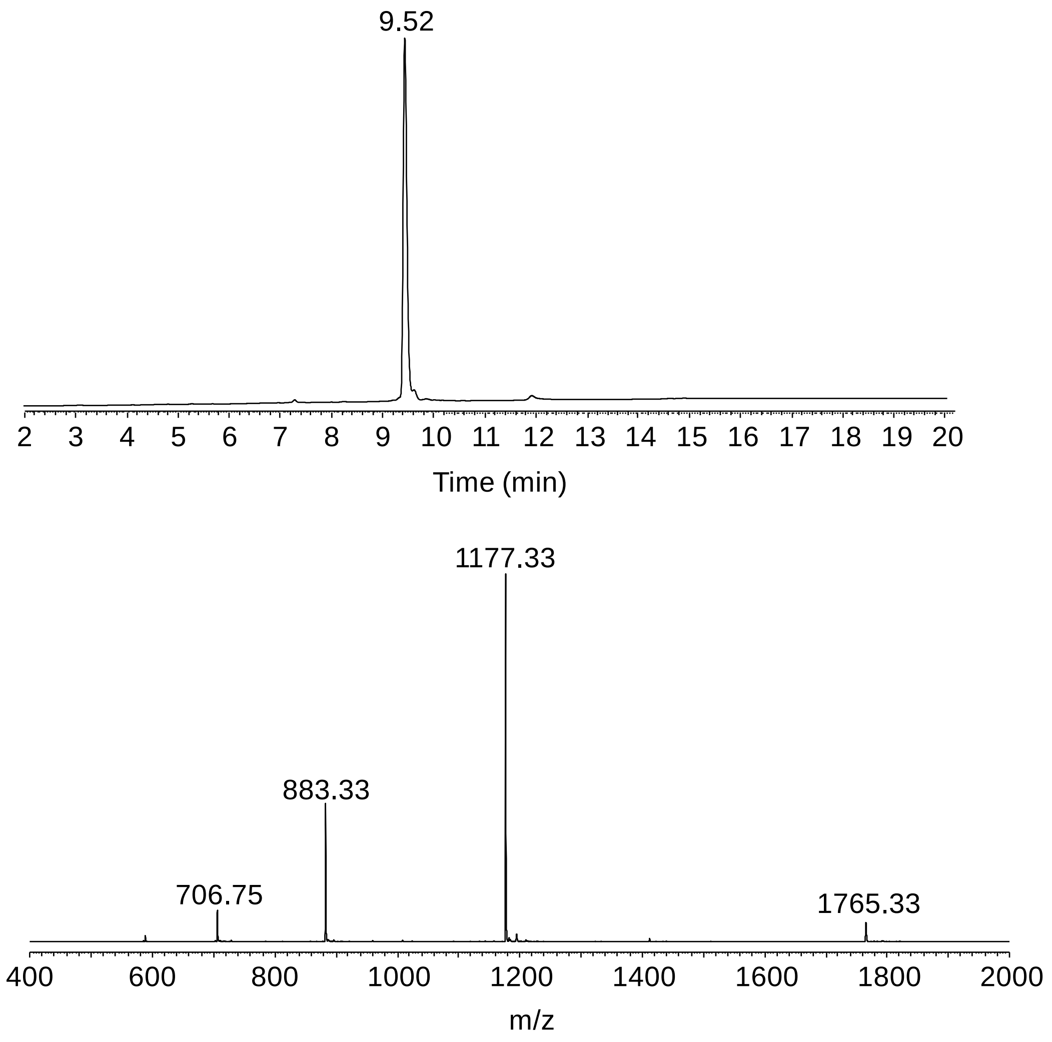

Figure S 3. UPLC-MS analysis of CRNFMRRYQSRVTQGLVAGETAQQICEDLR-NH_2 peptide **22**. LC trace: eluent A 0.10% TFA in water, eluent B 0.10% TFA in CH_3CN. C18 column, gradient 0-70% B in 20 min, 50 °C, 0.4 mL min^{-1}, UV detection at 215 nm. MS trace: m/z 1765.33 ($[M+2H]^{2+}$), 1177.33 ($[M+3H]^{3+}$), 883.33 ($[M+4H]^{4+}$), 706.75 ($[M+5H]^{5+}$); calcd. for [M] (average mass): 3529.04, found: 3529.04.

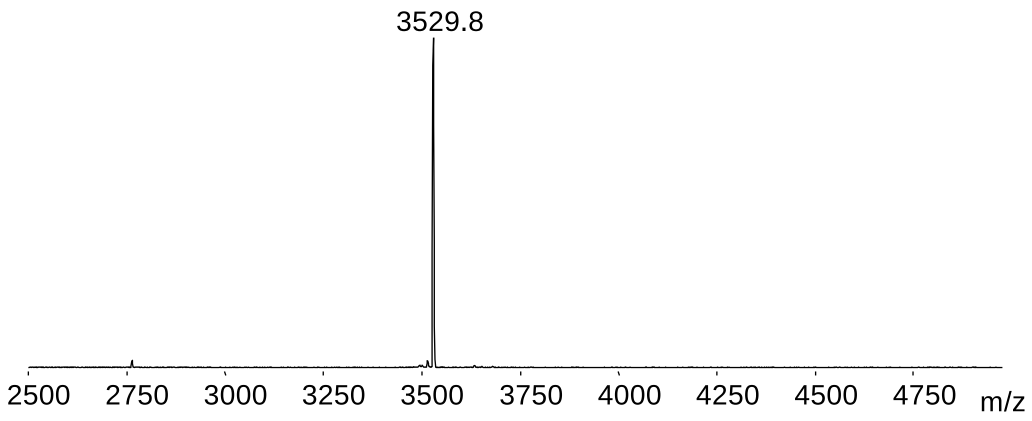

Figure S 4. MALDI-TOF analysis of peptide **22**. Matrix 2,5-dihydrobenzoic acid, positive detection mode, calcd. for $[M+H]^+$ (average): 3530.04, found: 3529.8.

3.3. Synthesis and characterization of SEA^{off} peptides

C(S*t*Bu)LTIVQKLKKMVDKPTQRSVSNAATRVC(S*t*Bu)RTGRSRWRDV-SEA^{off} peptide **26** was synthesized on a 0.10 mmol scale as described in the general procedure starting from SEA ChemMatrix® beads until reaching Arg 13. At that point, the coupling of the Fmoc-Ala-Thr(Ψ(Me,Me)Pro-OH dipeptide unit was performed manually as following. To a solution of Fmoc-Ala-Thr(Ψ(Me,Me)Pro-OH (91 mg, 0.20 mmol, 2.0 equiv.) in DMF (2.0 mL) were successively added HATU (74 mg, 0.19 mmol, 1.9 equiv.) and DIEA (70 µL, 0.40 mmol, 4.0 equiv.) and the mixture was stirred at rt for 2 min. The preactivated dipeptide unit was then added to the beads (0.10 mmol, 1.0 equiv.) swelled in the minimal volume of DMF. The beads were agitated at rt for 1 h and washed with DMF (3 × 2 min). The coupling was repeated a second time and a capping step was then performed using Ac_2O/DIEA/DMF 10/5/85 v/v/v (2 × 5 min). The beads were washed with DMF (3 × 2 min) and then treated with DMF/piperidine 80/20 v/v (2 × 10 min) to remove the Fmoc protecting group. Finally, the solid support was washed with DMF (3 × 2 min) and the automated peptide elongation was reruned from Ala 16 to the final Cys residue. TFA/EDT/TIS/thiophenol/thioanisole 87.5/2.5/5/2.5/2.5 v/v/v/v/v (10 mL) was used as the cleavage cocktail. Purification of the crude by preparative HPLC (eluent A 0.10% TFA in water, eluent B 0.10% TFA in CH_3CN/water 4/1 v/v, 0-32% eluent B in 2 min then 32-44% in 30 min, 50 °C, detection at 215 nm, 6 mL min^{-1}, C18 Xbridge 5 µm (10 × 250 mm) column) provided peptide **26** as a white solid after lyophilisation (97.2 mg, 17%).

Characterization of C(StBu)LTIVQKLKKMVDKPTQRSVSNAATRVC(StBu)RTGRSRWRDV-SEA^off peptide **26**

Peptide **26** was analyzed by UPLC-MS (Figure S 5) and MALDI-TOF mass spectrometry (Figure S 6).

ESI (positive detection mode, see Figure S 5): calcd. for [M] (average): 4682.77, found: 4682.95.

MALDI-TOF (positive detection mode, matrix 2,5-dihydrobenzoic acid, see Figure S 6): calcd. for $[M+H]^+$ (average): 4683.77, found: 4684.22.

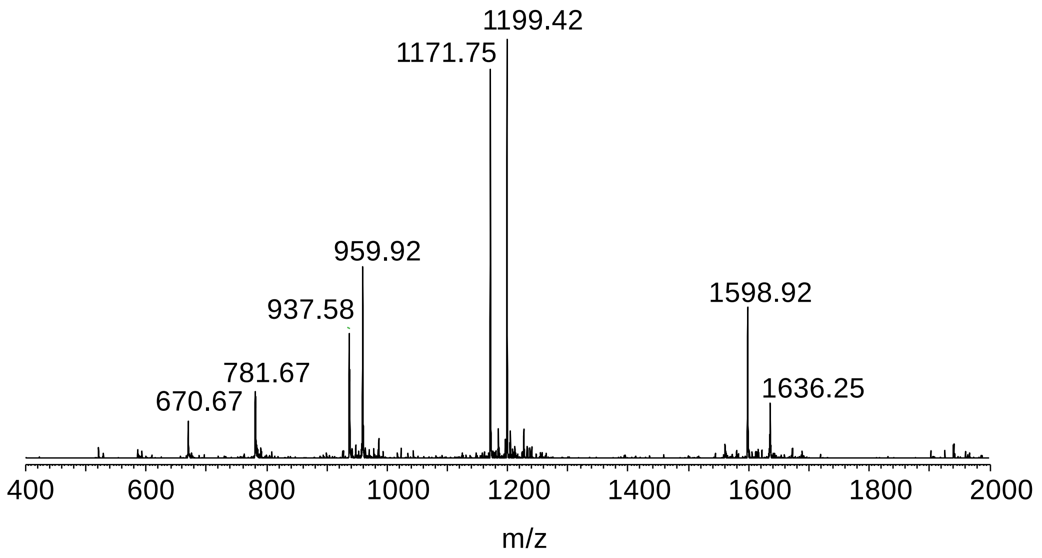

Figure S 5. UPLC-MS analysis of C(S*t*Bu)LTIVQKLKKMVDKPTQRSVSNAATRVC(S*t*Bu)-RTGRSRWRDV-SEA^off peptide **26**. LC trace: eluent A 0.10% TFA in water, eluent B 0.10% TFA in CH_3CN. C18 column, gradient 0-70% B in 20 min, 50 °C, 0.4 mL min^{-1}, UV detection at 215 nm. MS trace: m/z 1636.25 ($[M+2M_{TFA}+3H]^{3+}$), 1598.92 ($[M+M_{TFA}+3H]^{3+}$), 1199.42 ($[M+M_{TFA}+4H]^{4+}$), 1171.75 ($[M+4H]^{4+}$), 959.92 ($[M+M_{TFA}+5H]^{5+}$), 937.58 ($[M+5H]^{5+}$), 781.67 ($[M+6H]^{6+}$), 670.67 ($[M+7H]^{7+}$); calcd. for [M] (average mass): 4682.77, found: 4682.95.

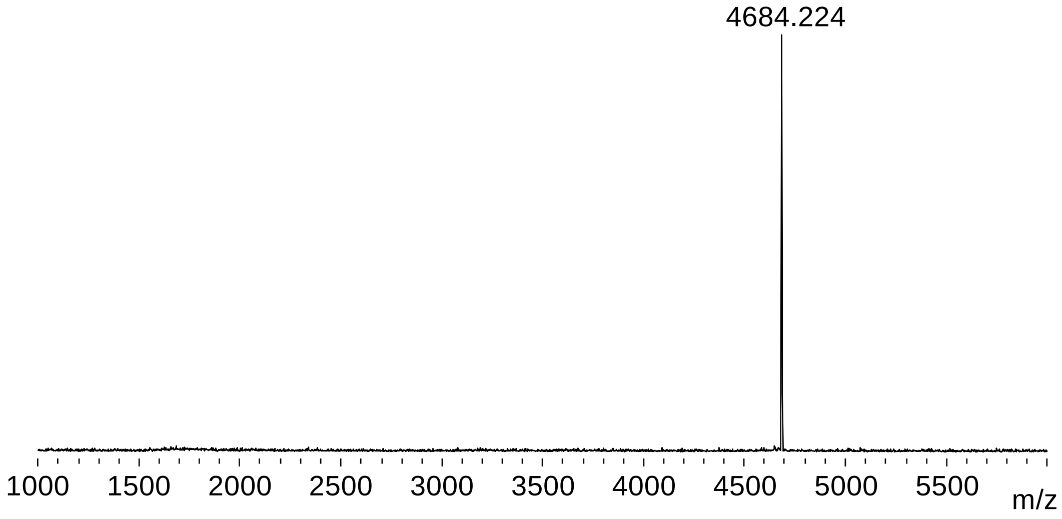

Figure S 6. MALDI-TOF analysis of peptide **26**. Matrix 2,5-dihydrobenzoic acid, positive detection mode, calcd. for $[M+H]^+$ (average): 4683.77, found: 4684.22.

DYRT-SEA^{off} peptide was synthesized on a 0.10 mmol scale as described in the general procedure starting from SEA PS beads. TFA/EDT/TIS/thiophenol/thioanisole 87.5/2.5/5/2.5/2.5 v/v/v/v/v (20 mL) was used as the cleavage cocktail. Purification of the crude by semi-preparative HPLC (eluent A 0.10% TFA in water, eluent B 0.10% TFA in CH_3CN, 20 mL min^{-1}, 0-10% eluent B in 10 min and then 10-30% eluent B in 45 min, C18 XBridge 5 µm (19 × 250 mm) column, detection at 215 nm, 20 °C) provided DYRT-SEA^{off} peptide as a white solid after lyophilisation (45.0 mg, 50%).

Characterization of DYRT-SEA^{off} peptide

The DYRT-SEA^{off} peptide was analyzed by UPLC-MS (Figure S 7) and MALDI-TOF mass spectrometry (Figure S 8).
ESI (positive detection mode, see Figure S 7): calcd. for $[M+H]^+$ (monoisotopic): 671.26, found: 671.42.
MALDI-TOF (positive detection mode, matrix 2,5-dihydrobenzoic acid, Figure S 8): calcd. for $[M+H]^+$ (monoisotopic): 671.26, found: 671.15.

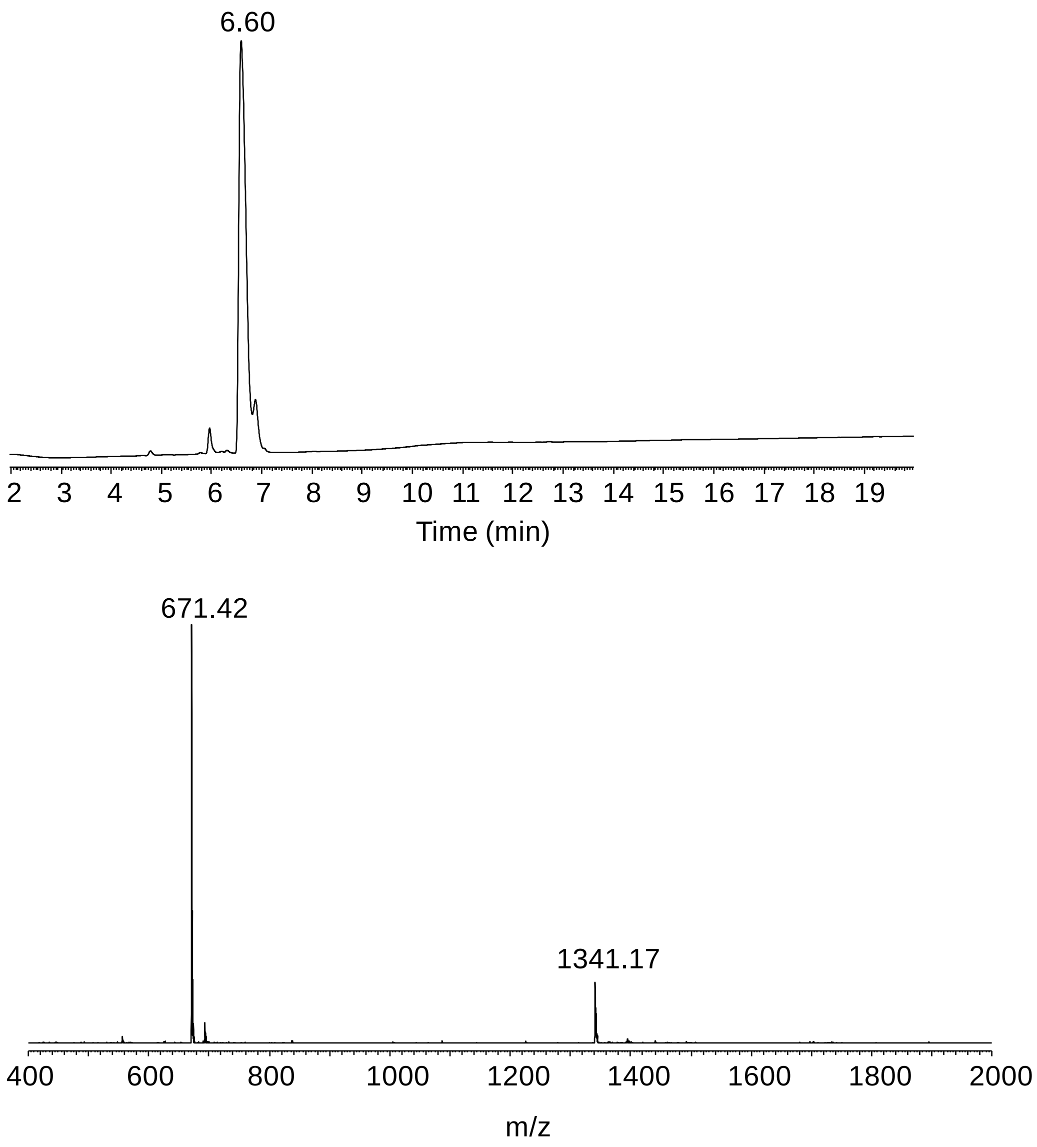

Figure S 7. UPLC-MS analysis of DYRT-SEA^{off} peptide. LC trace: eluent A 0.10% TFA in water, eluent B 0.10% TFA in CH_3CN. C18 column, gradient 0-70% B in 20 min, 50 °C, 0.4 mL min^{-1}, UV detection at 215 nm. MS trace: m/z 671.42 ([M+H]$^+$); calcd. for [M+H]$^+$ (monoisotopic): 671.26, found: 671.42.

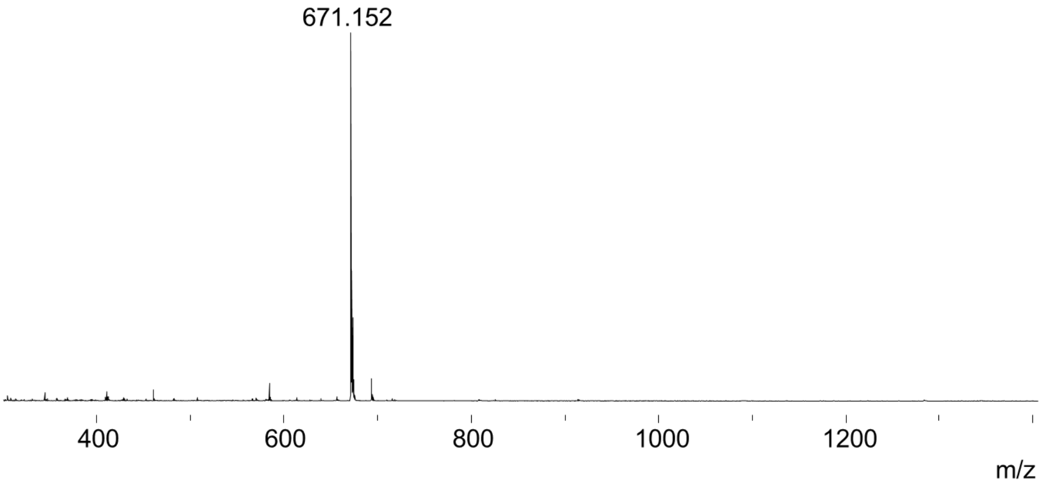

Figure S 8. MALDI-TOF analysis of DYRT-SEA^{off} peptide. Matrix 2,5-dihydrobenzoic acid, positive detection mode, calcd. for $[M+H]^+$ (monoisotopic): 671.26, found: 671.15.

3.4. Synthesis and characterization of MPA thioester peptides

DYRT-MPA peptide **24** was synthesized by converting the SEA^{off} peptide into the corresponding MPA thioester using the following procedure.

MPA (0.15 mL, 5%-vol), TCEP·HCl (0.17 g, 0.59 mmol) were dissolved in 3.0 mL of 6 M Gn·HCl aqueous solution. SEA^{off} peptide (10 mg, 15 µmol) was dissolved in 3.0 mL of the former solution, pH was adjusted to 4.0 by addition of 6 M NaOH and the reaction mixture was stirred under nitrogen atmosphere at 37 °C for 24 h. Purification of the crude by semi-preparative HPLC (eluent A 0.10% TFA in water, eluent B 0.10% TFA in CH_3CN/water 4/1 v/v, 6 mL min^{-1}, 0-20% eluent B in 35 min, C18 XBridge 5 µm (10 × 250 mm) column, detection at 215 nm, 50 °C) provided DYRT-MPA peptide **24** as a white solid after lyophilization (8.33 mg, 64%).

Characterization of DYRT-MPA peptide **24**

Peptide **24** was analyzed by UPLC-MS (Figure S 9) and MALDI-TOF mass spectrometry (Figure S 10). ESI (positive detection mode, see Figure S 9): calcd. for $[M+H]^+$ (monoisotopic): 642.25, found: 642.42.
MALDI-TOF (positive detection mode, matrix 2,5-dihydrobenzoic acid, Figure S 10): calcd. for $[M+H]^+$ (monoisotopic): 642.25, found: 642.18.

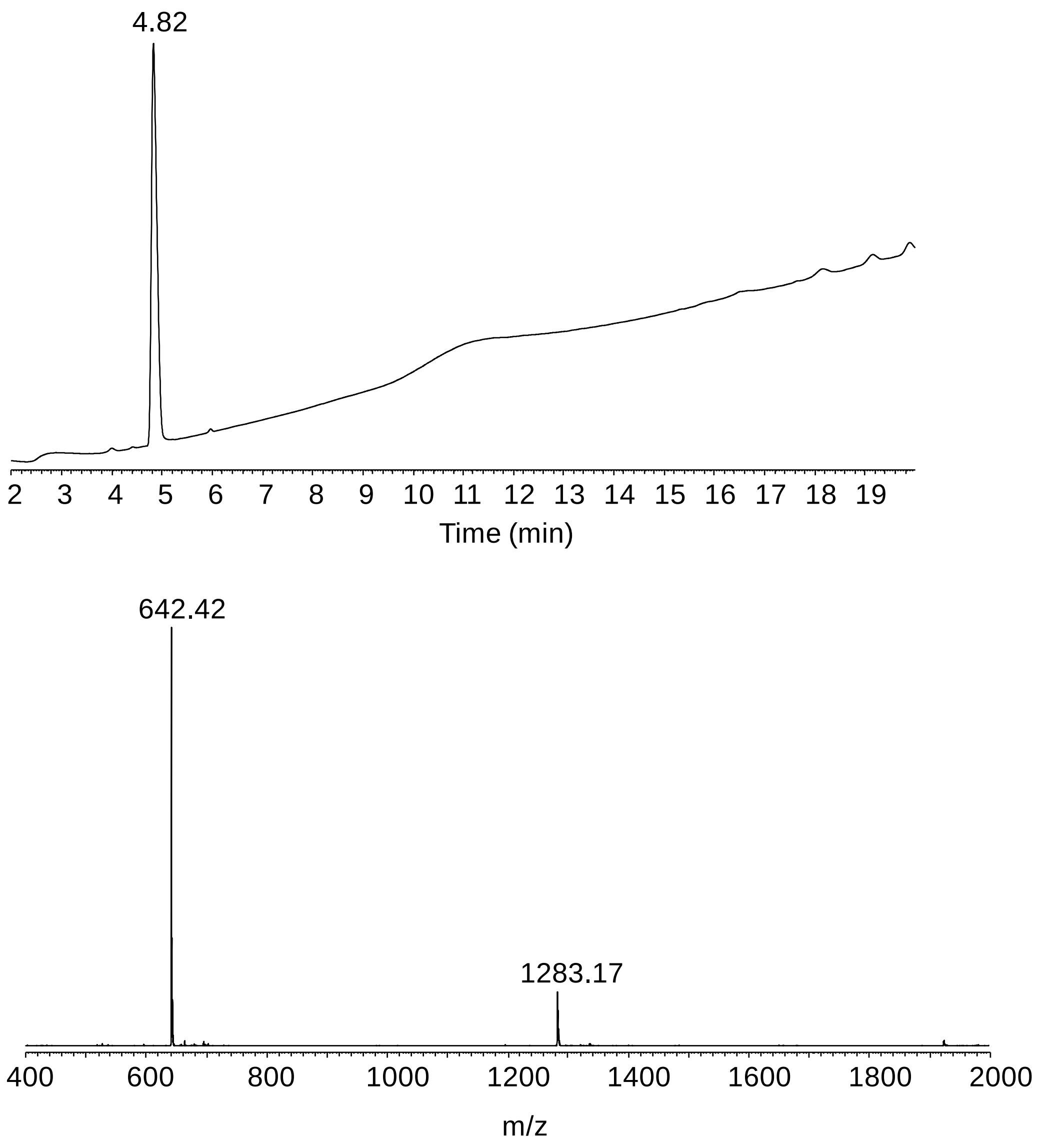

Figure S 9. UPLC-MS analysis of DYRT-MPA peptide **24**. LC trace: eluent A 0.10% TFA in water, eluent B 0.10% TFA in CH_3CN. C18 column, gradient 0-70% B in 20 min, 50 °C, 0.4 mL min^{-1}, UV detection at 215 nm. MS trace: m/z 1283.17 ($[2M+H]^+$), 642.42 ($[M+H]^+$); calcd. for $[M+H]^+$ (monoisotopic): 642.25, found: 642.42.

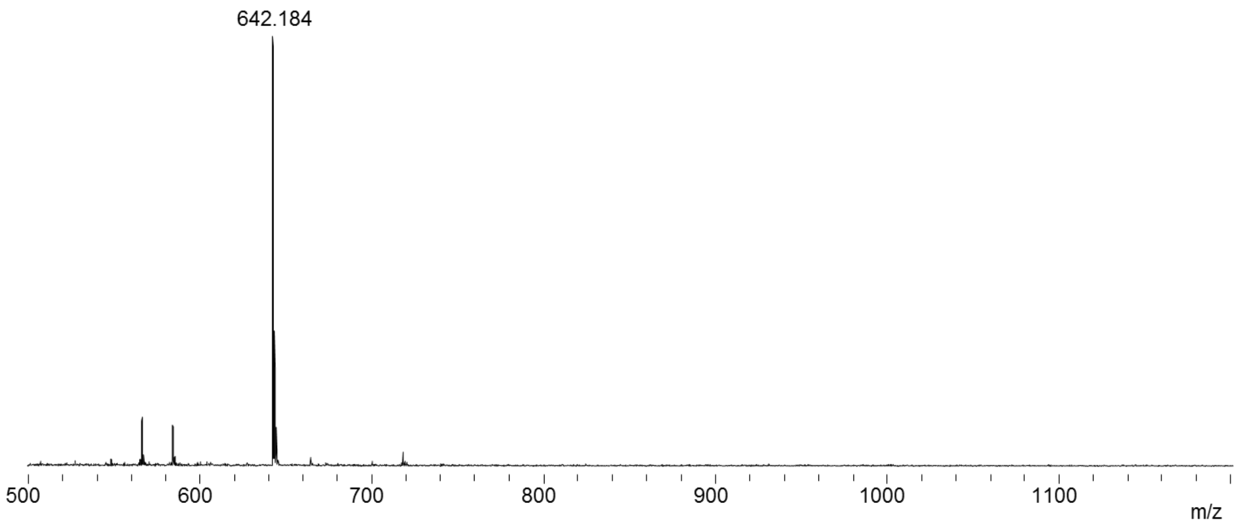

Figure S 10. MALDI-TOF analysis of peptide **24**. Matrix 2,5-dihydrobenzoic acid, positive detection mode, calcd. for $[M+H]^+$ (monoisotopic): 642.25, found: 642.18.

4. Synthesis of diselenide precatalysts

4.1. Diselenide precatalyst 17

Diselenide precatalyst **17** was prepared as described below in Figure S 11.

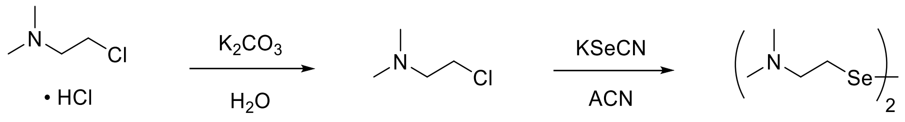

17

Figure S 11. Synthesis scheme for the preparation of diselenide precatalyst **17**.

*Characterization of **17***

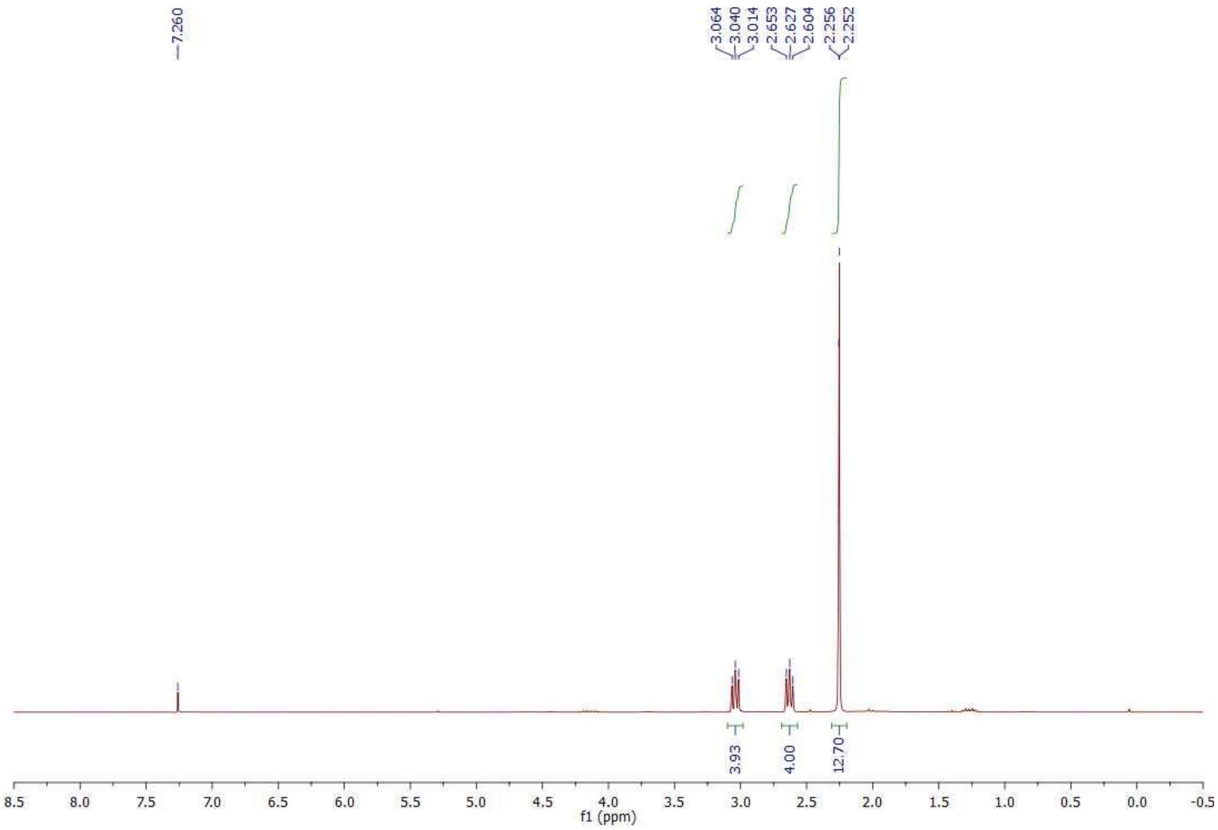

Figure S 12. ^{1}H NMR (300 MHz) spectrum of compound **17** ($CDCl_3$, 293 K)

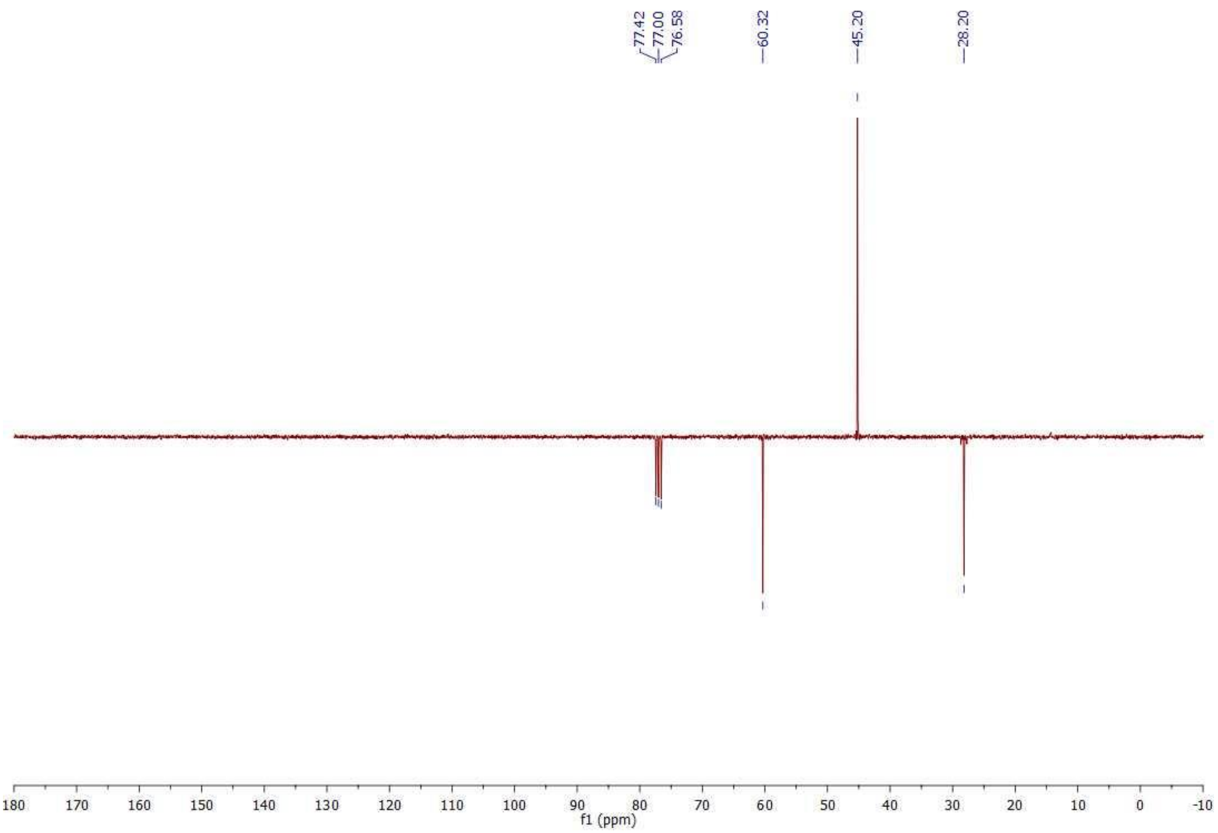

Figure S 13. ^{13}C APT NMR (75 MHz) spectrum of compound **17** ($CDCl_3$, 293 K).

4.2. Diselenide precatalyst 18

Bis(2-dimethylaminoethyl)diselenide **17** (600 mg, 1.99 mmol) was dissolved in 40 mL of diethyl ether/acetone 1/1 (v/v). A methyl iodide solution (396 µL, 6.41 mmol) was slowly added and the reaction mixture was stirred 2 h at room temperature. The crude precipitate was collected by filtration and washed 3 times with 10 mL of cold diethyl ether/acetone 1/1 (v/v).

The crude solid diselenide **18** was dried overnight under vaccum and then recrystallized from methanol to provide diselenide **18** as yellow crystals (462 mg, 0.788 mmol, 40 %).

^{1}**H NMR** (300 MHz, D_2O) δ (ppm) 3.71 – 3.66 (m, 4H), 3.30 – 3.25 (m, 4H), 3.12 (s, 18H) (see Figure S 14). ^{13}**C NMR** (75 MHz, D_2O) δ (ppm) 67.63, 52.85, 19.62 (see Figure S 15).

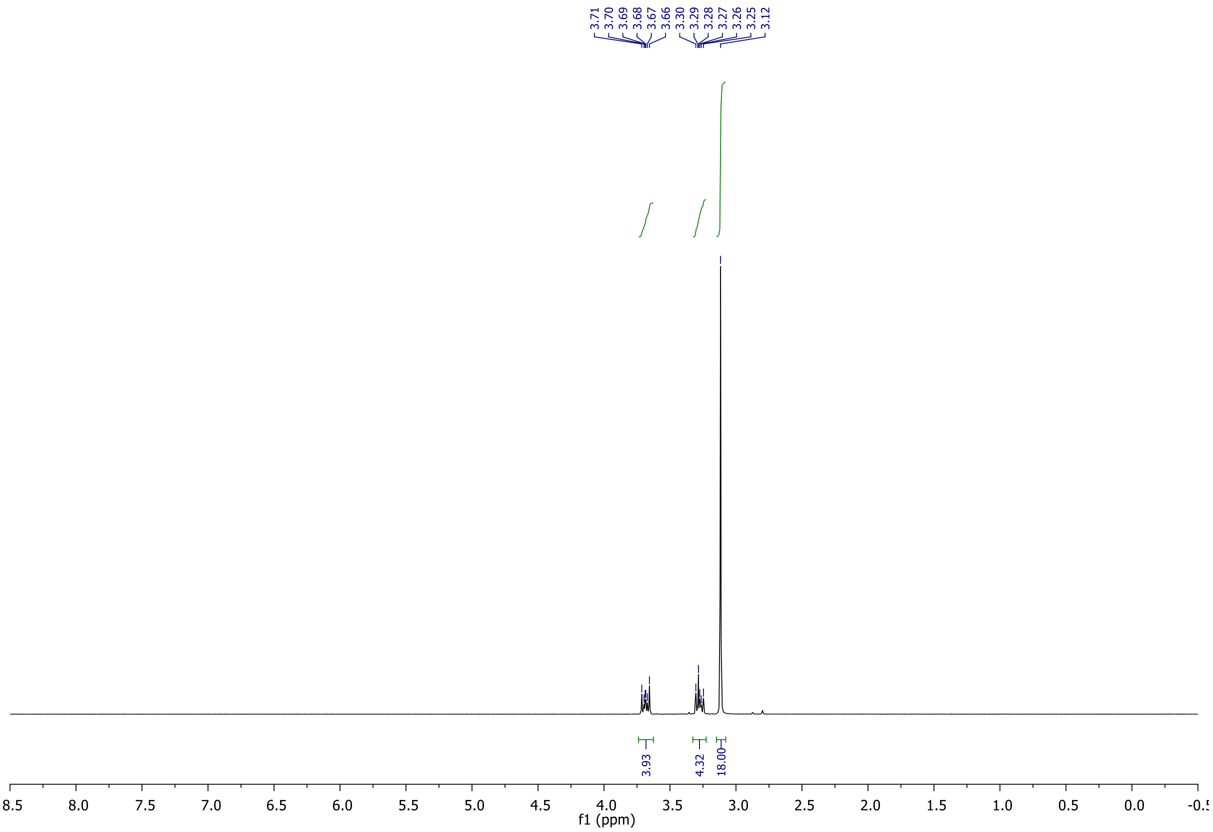

Figure S 14. ^{1}H NMR (300 MHz) spectrum of compound **18** (D_2O, 293 K).

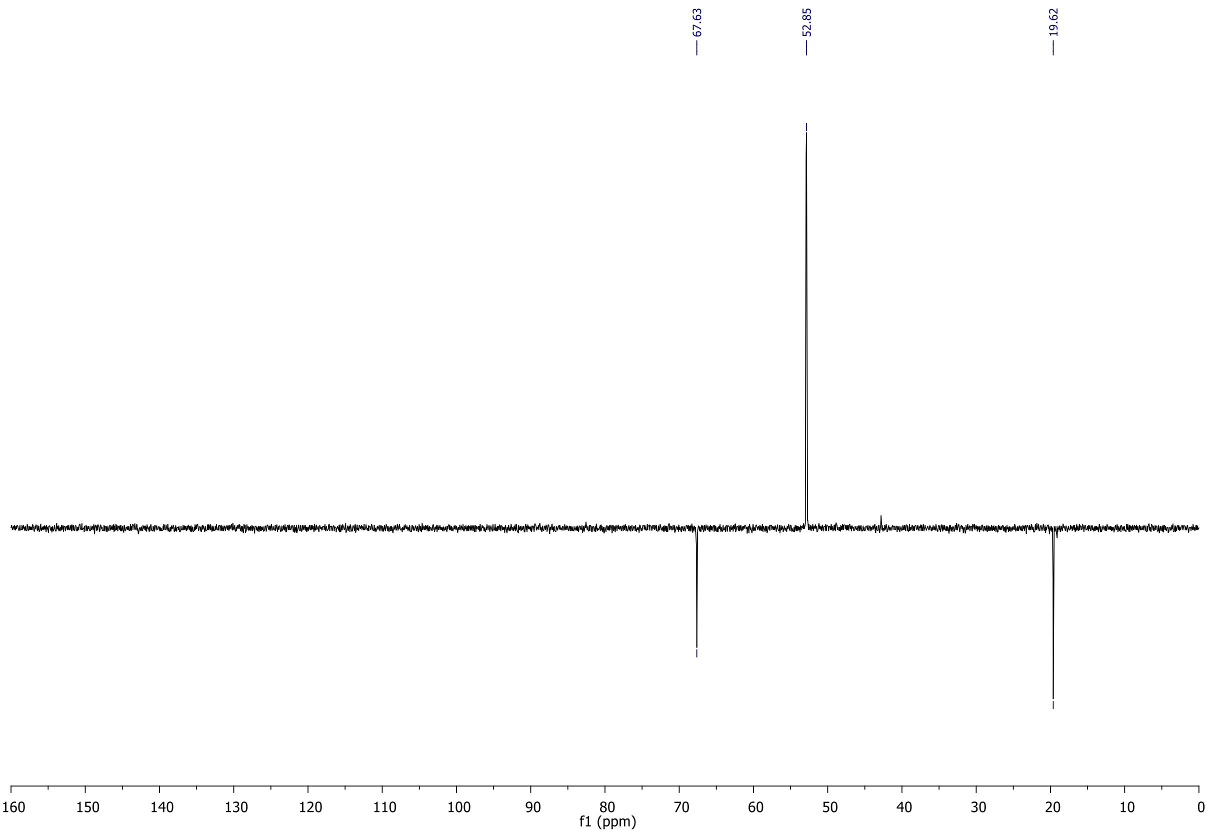

Figure S 15. ^{13}C APT NMR (75 MHz) spectrum of compound **18** (D_2O, 293 K).

1. Kinetic studies and modelisation

5.1. Generalities

Kintek Global Kinetic Explorer Software (version 10.0.200514) was used for kinetic modelization. The model reaction used for numerical fitting corresponds to a second-order reaction in the form of A + B $\rightarrow$ C.

The standard deviation for each trace was first estimated upon fitting the experimental dataset with an analytical function (3-exponential) so as to determine an average sigma value further used for numerical data fitting. The subsequent numerical fit allowed determining the apparent second order rate constant k_{app} for each experiment. Fitting to a given model was achieved by nonlinear regression analysis based upon an iterative search to find a set of reaction parameters that gives a minimum χ^2. The process was completed by careful visual examination of the fits.

5.2. Rate constants of uncatalyzed and catalyzed SEA peptide / MPA exchange reactions

Experimental procedure

The general procedure for studying the effect of the catalyst and catalyst concentration on the rate of thiol-thioester exchange is illustrated with the following conditions: 1 mM SEA-peptide, 6 M Gn·HCl, 100 mM diselenide precatalyst **17**, 100 mM TCEP, 3-mercaptopropionic acid (5% v/v), pH 4.0 in 0.1 M phosphate buffer at 37 °C.

To a solution of TCEP·HCl (28.67 mg, 100 µmol, 100 mM final concentration) in 6 M Gn·HCl, 0.1 M, pH 7.2 sodium phosphate buffer (1 mL) was added the alkyldiselenide precatalyst **17** (30.2 mg, 0.1 mmol, 100 mM final concentration). To the resulting mixture was added MPA (5% v/v). Finally, the pH was adjusted to 4.0 by addition of 6 M NaOH and the SEA peptide (1.42 mg, 1.0 µmol) was dissolved in the previous solution. The reaction mixture was stirred at 37 °C under nitrogen atmosphere. The progress of the reaction was monitored by HPLC. For each point, a 2 µL aliquot was taken from the reaction mixture and quenched by addition of 50 µL of 10% aqueous AcOH. The chromatograms were processed on the basis of the absorbance signal at 215 nm to deduce the conversion ratio of the different species (no absorbance correction was applied as SEA peptides and MPA thioester were considered to have the same molecular extinction coefficient).

Results

Table S 1. Kinetic data extracted from curve fitting.[a]

Catalyst (Precatalyst)	Total selenol or thiol concentration (mM)	$k_{app} \times 10^{-3}$ ($M^{-1}.min^{-1}$)	StdErr $\times 10^{-3}$ ($M^{-1}.min^{-1}$)
-	-	2.68	0.56
**MPAA**[b]	100	4.52	2.65
**8a (7a)**	200	10.22	1.28
**13 (17)**	200	11.9	1.94
**13 (17)**	100	9.67	0.49
**13 (17)**	50	9.07	0.92
**13 (17)**	25	6.27	0.51
**13 (17)**	12.5	4.36	0.78
**13 (17)**	6.25	3.42	0.38

[a] General conditions are as follows: 1 mM SEA-peptide, 6 M Gn·HCl, catalyst, 100 mM TCEP, 3-mercaptopropionic acid (5% v/v), pH 4.0 in 0.1 M phosphate buffer at 37 °C. Rate constants were extracted using KinteK Explorer Software™. A standard deviation estimate for experimental measurements was obtained from fitting with a multi-exponential function. Rate constants and associated standard errors were produced by chi² minimization fitting. Standard errors were estimated based upon the covariance matrix using Kintek software.

[b] Raw data with MPAA used as catalyst were extracted from reference 47 (*J. Org. Chem.* **2018**, *83*, 12584–12594).

Table S 2. Fitting of MPA thioester formation in the presence of catalysts.[a]

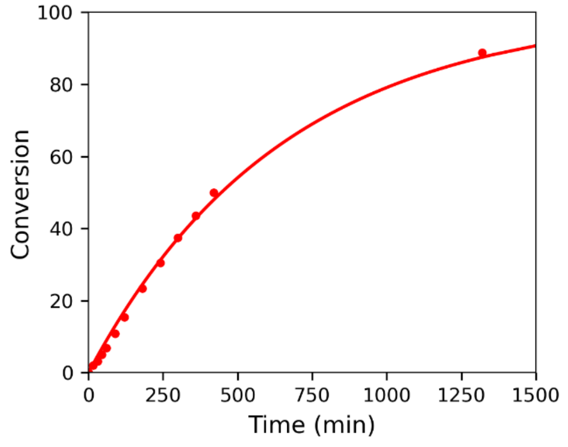

Uncatalyzed

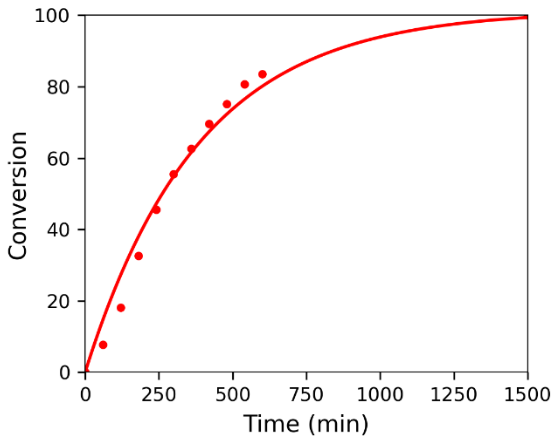

100 mM MPAA[b]

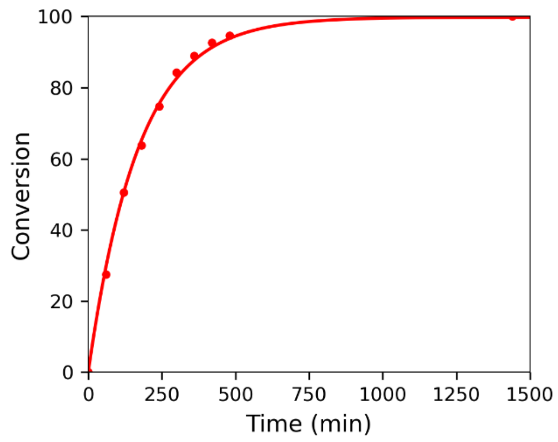

200 mM catalyst **8a**

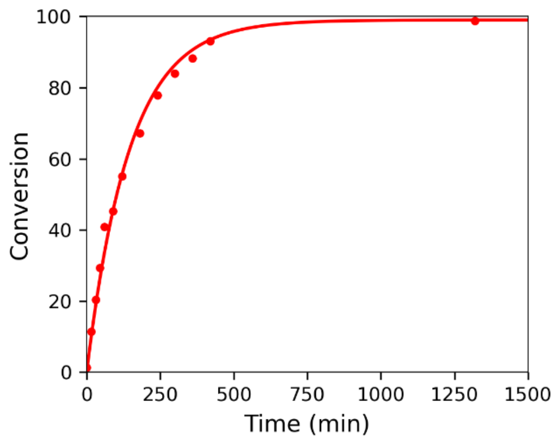

200 mM catalyst **13**

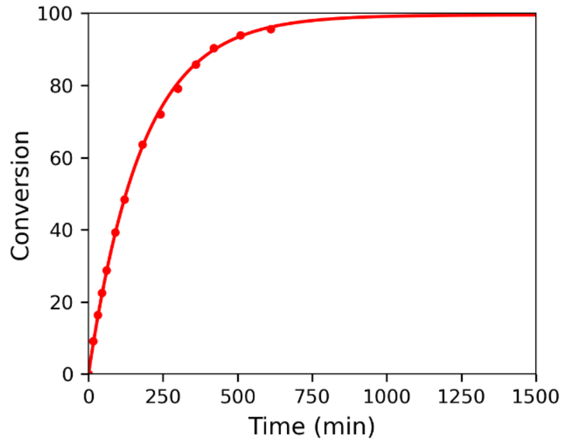

100 mM catalyst **13**

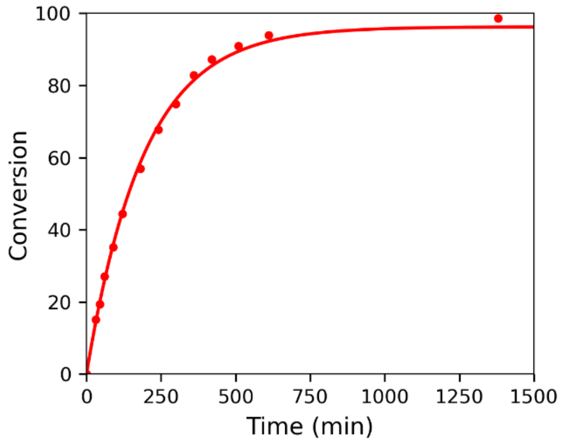

50 mM catalyst **13**

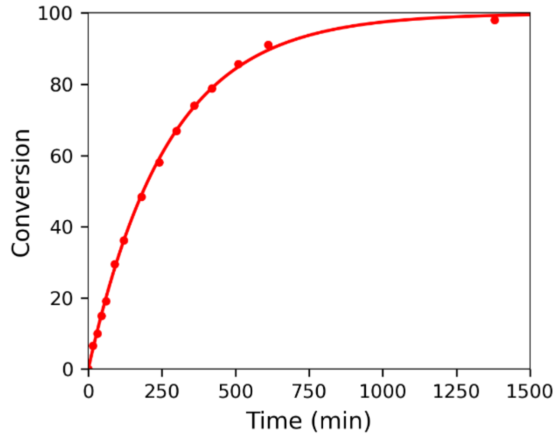

25 mM catalyst **13**

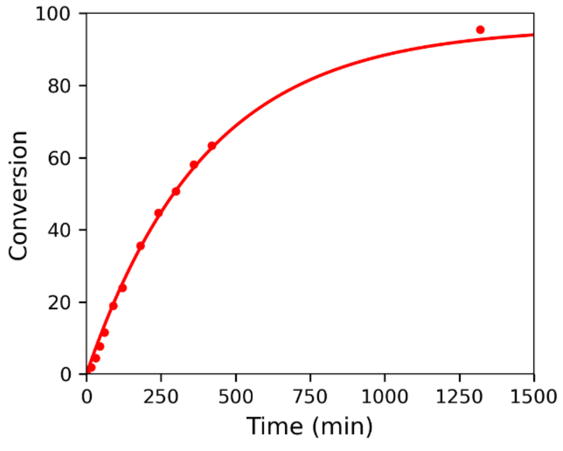

12.5 mM catalyst **13**

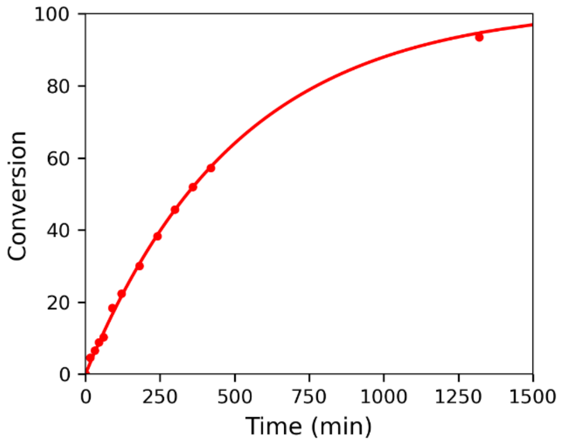 6.25 mM catalyst **13**	

[a] The dots correspond to experimental values. The continuous line corresponds to the fitting curve.

[b] Raw data with MPAA used as catalyst were extracted from reference 47 (*J. Org. Chem.* **2018**, *83*, 12584–12594).

5.3 Analytical approach

Data fitting based on numerical approaches (as proposed above) does not involve translating the problem into an analytical expression. Alternatively, the problem could be formulated analytically with respect to standard equations for a pseudo-first order kinetics. In this case, MPA is being considered as the reagent in large excess.

$$\text{Conversion of Product in \%age} = ([A]_0 - [A]_0.e^{-kt})*100/[A]_0 \qquad (1)$$

with $[A]_0$ initial peptide concentration (M), k = pseudo-first order rate constant (min^{-1}), time (min)

We can verify that experimental data can be fitted to this equation (1). The red plots in the chart below represent the experimental datapoints for catalyst **13** used at 200 mM. The fitting curve (dashed line) was traced using equation (1) with a value of $[A]_0 = 0.001$ M and $k = 0.0119 \times 0.574 = 0.00686$ min^{-1}.

The fit obtained (see Figure S 16) is perfectly consistent with the corresponding data presented in table S2 and obtained from the numerical approach.

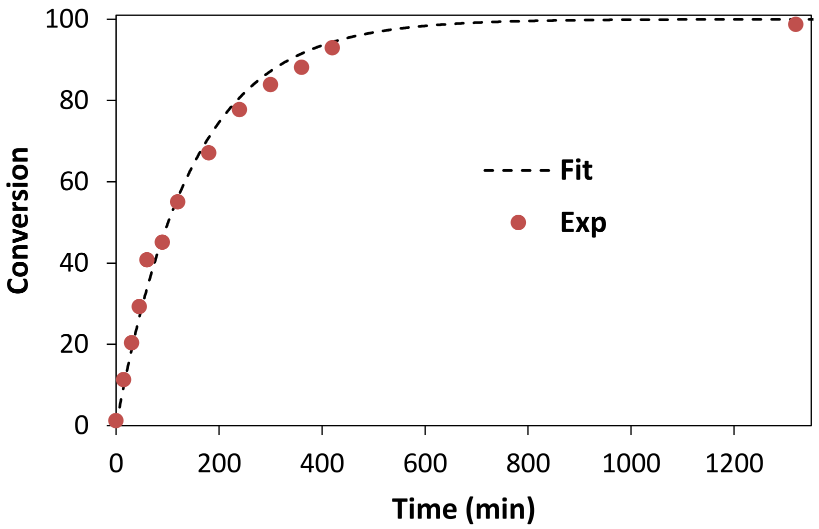

Figure S 16. Fitting of SEA/MPA exchange in the presence of catalyst **13** (200 mM). Data were fitted according to equation (1).

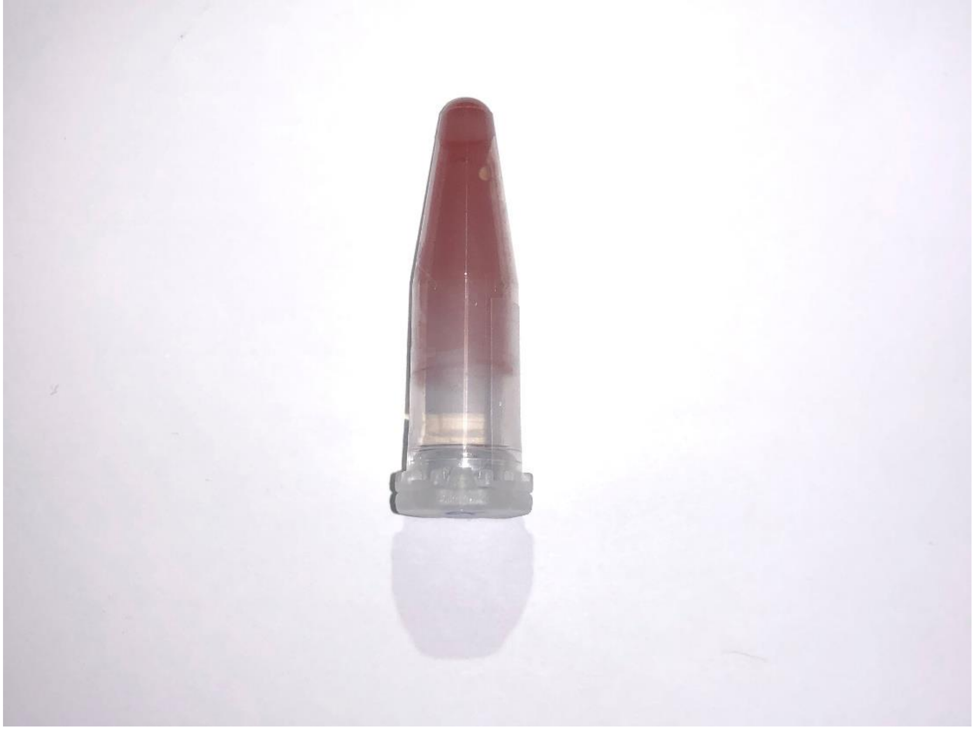

Figure S 17. Red selenium precipitate formed during catalysis of SEA/MPA exchange in the presence of catalyst **14** (200 mM).

2. Synthesis of linear 9 kDa granulysin polypeptide 9-GN-l

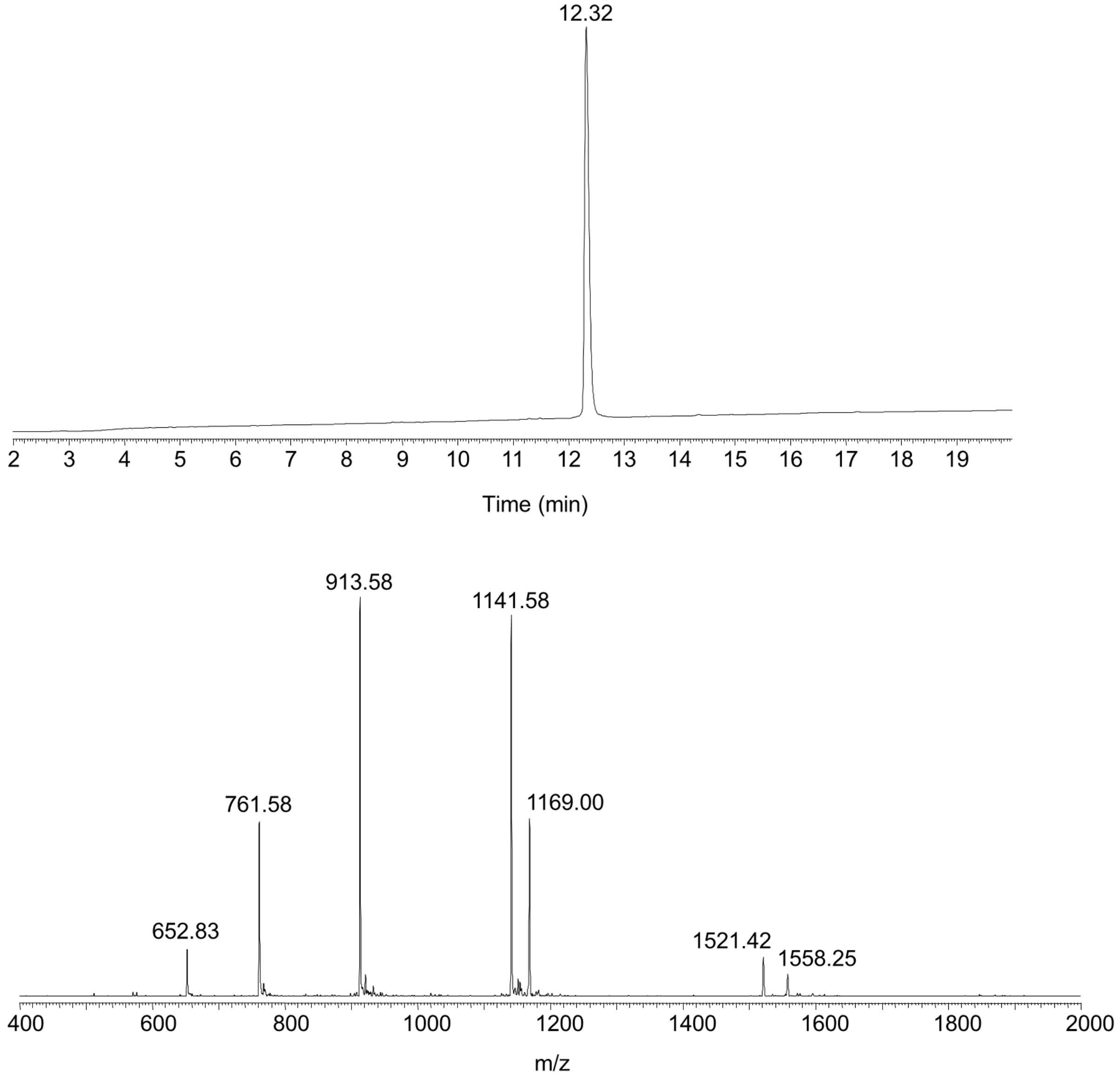

Figure S 18. UPLC-MS analysis of peptide **21**. LC trace: eluent A 0.10% TFA in water, eluent B 0.10% TFA in CH_3CN. C18 column, gradient 0-70% B in 20 min, 50 °C, 0.4 mL min^{-1}, UV detection at 215 nm. MS trace: m/z 1558.25 ([M+M_{TFA}+3H]$^{3+}$), 1521.42 ([M+3H]$^{3+}$), 1169.00 ([M+M_{TFA}+4H]$^{4+}$), 1141.58 ([M+4H]$^{4+}$), 913.58 ([M+5H]$^{5+}$), 761.58 ([M+6H]$^{6+}$), 652.83 ([M+7H]$^{7+}$); calcd. for [M] (average mass): 4561.40, found: 4561.79.

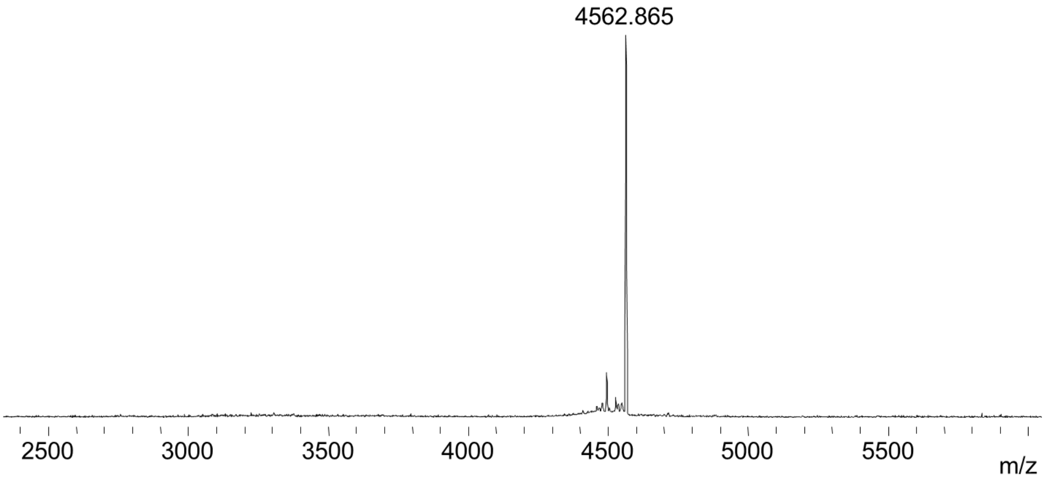

Figure S 19. MALDI-TOF analysis of peptide **21**. Matrix 2,5-dihydrobenzoic acid, positive detection mode, calcd. for $[M+H]^+$ (average): 4562.40, found: 4562.87.

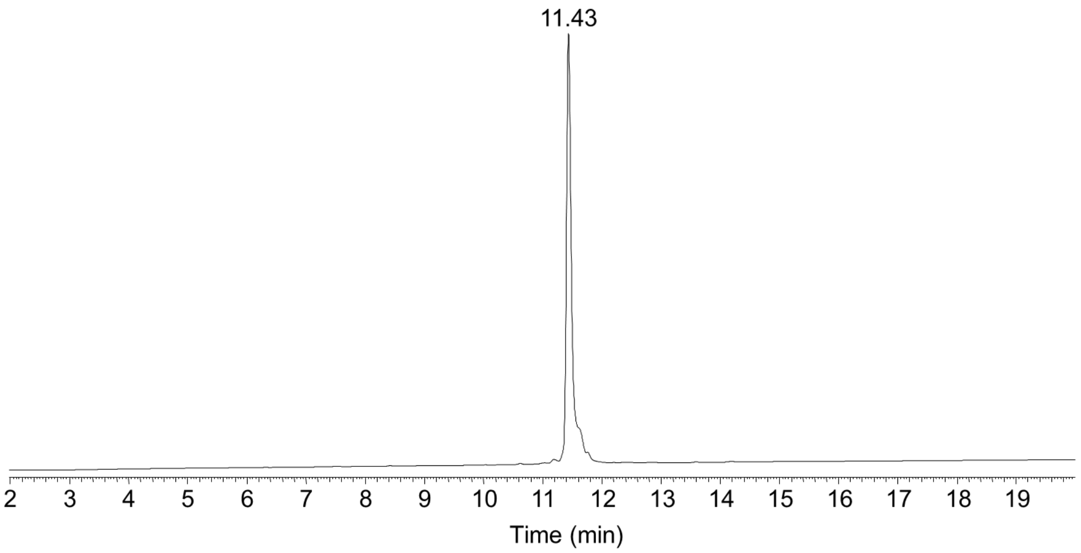

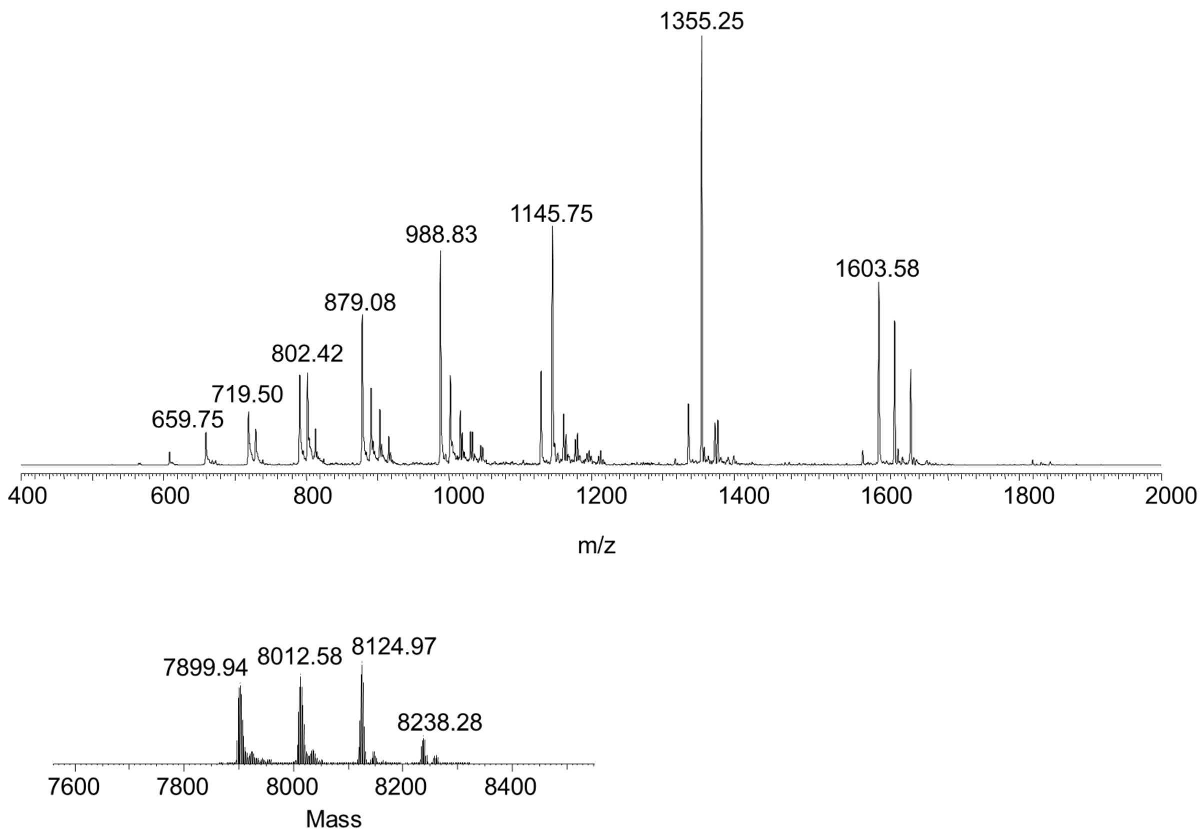

Figure S 20. UPLC-MS analysis of peptide **23**. LC trace: eluent A 0.10% TFA in water, eluent B 0.10% TFA in CH_3CN. C18 column, gradient 0-70% B in 20 min, 50 °C, 0.4 mL min^{-1}, UV detection at 215 nm. MS trace: calcd. for [M] (average mass): 7899.22, found: 7899.94 (8012.58 [M+M_{TFA}], 8124.97 [M+2M_{TFA}], 8238.28 [M+3M_{TFA}]).

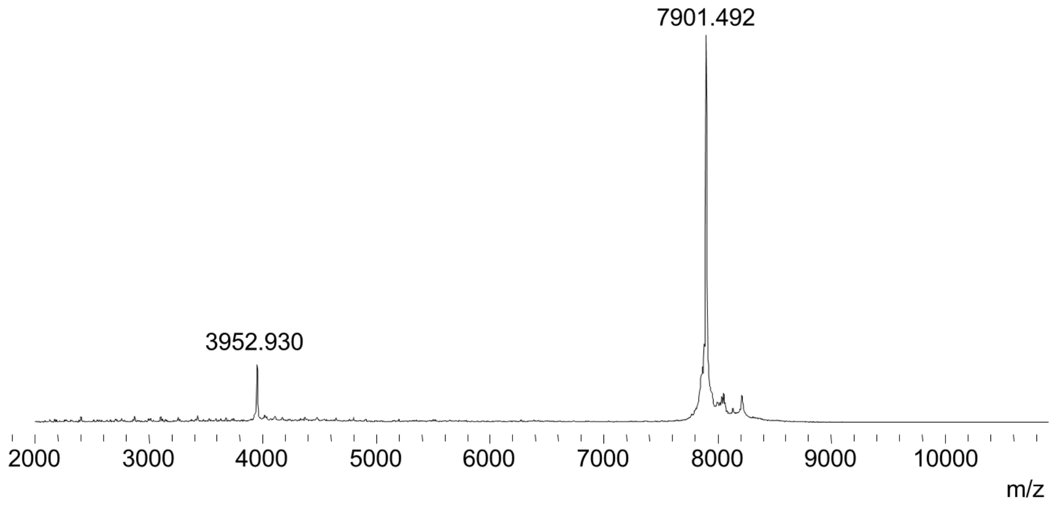

Figure S 21. MALDI-TOF analysis of peptide **23**. Matrix 2,5-dihydrobenzoic acid, positive detection mode, calcd. for $[M+H]^+$ (average): 7900.22, found: 7901.49.

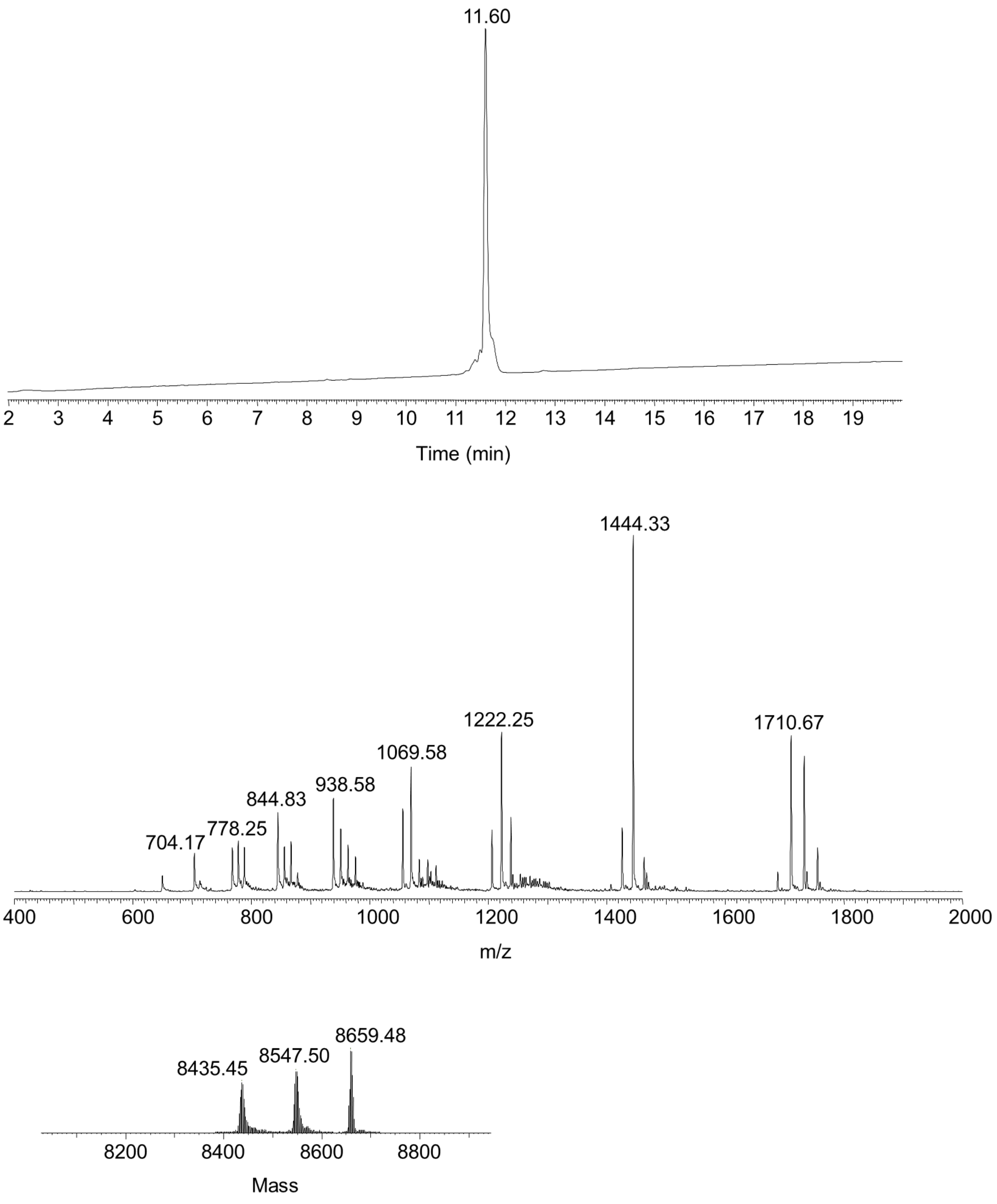

Figure S 22. UPLC-MS analysis of peptide **9-GN-l**. LC trace: eluent A 0.10% TFA in water, eluent B 0.10% TFA in CH_3CN. C18 column, gradient 0-70% B in 20 min, 50 °C, 0.4 mL min^{-1}, UV detection at 215 nm. MS trace: calcd. for [M] (average mass): 8435.79, found: 8435.45 (8547.50 [$M+M_{TFA}$], 8659.48 [$M+2M_{TFA}$]).

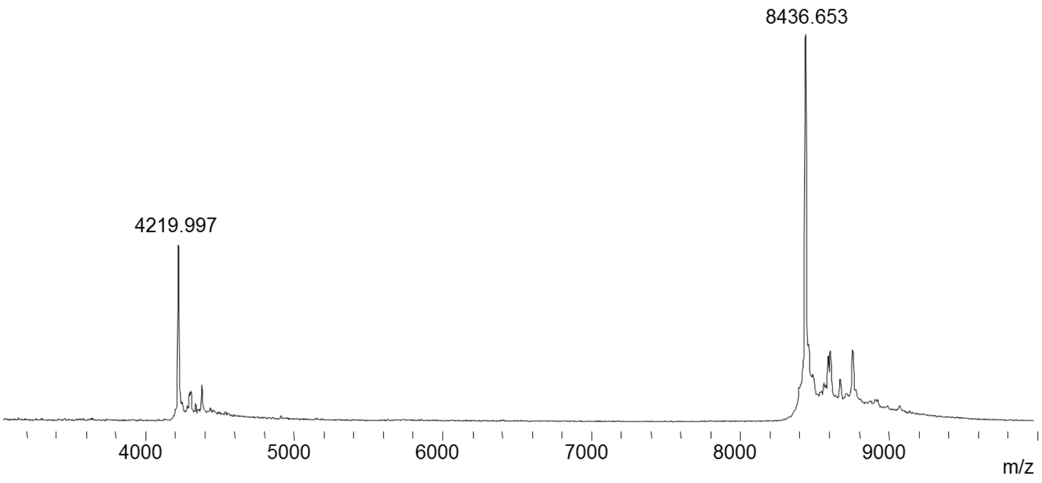

Figure S 23. MALDI-TOF analysis of peptide **9-GN-l**. Matrix 2,5-dihydrobenzoic acid, positive detection mode, calcd. for $[M+H]^+$ (average): 8436.79, found: 8436.65.

3. Folding of 9-GN-l and characterization of 9-GN

Characterization of folded 9-GN

Folded protein **9-GN** was analyzed by UPLC-MS (Figure S 24).

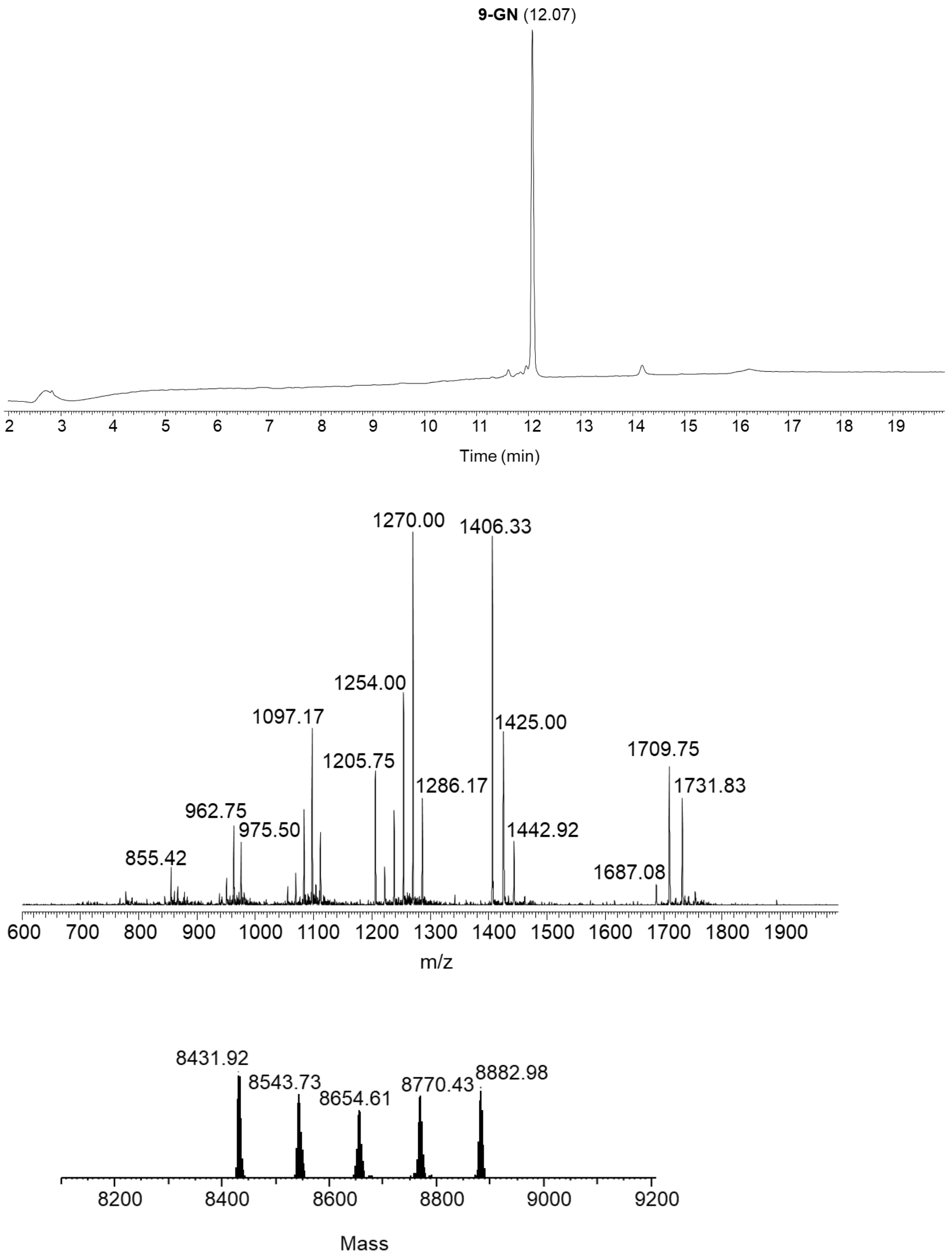

Figure S 24. UPLC-MS analysis of **9-GN**. LC trace: eluent A 0.10% TFA in water, eluent B 0.10% TFA in CH_3CN. C18 column, gradient 0-70% B in 20 min, 50 °C, 0.4 mL min^{-1}, UV detection at 215 nm. MS trace: calcd. for [M] (average mass): 8431.79, found: 8431.92 (8543.73 [$M+M_{TFA}$], 8654.61 [$M+2M_{TFA}$], 8770.43 [$M+3M_{TFA}$], 8882.98 [$M+4M_{TFA}$]).

Disulfide bridge pattern of 9-GN

The disulfide bridge patterns of **9-GN** is given in Figure S 25.

Experimental determination of the disulfide bridge pattern was achieved by trypsin digestion of **9-GN** and identification of the resulting fragments by mass spectrometry using non-reducing conditions. Fragments obtained by digestion with trypsin permitted the direct assignment of Cys69-Cys132 and Cys96-Cys107 disulfide bonds.

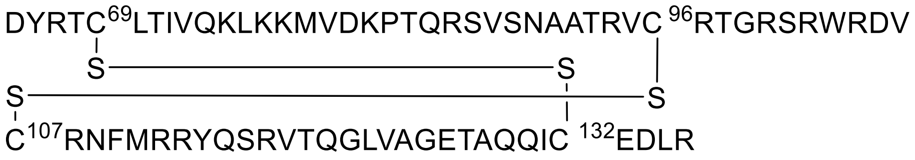

9-GN

Figure S 25. Disulfide bridge pattern of **9-GN**.

Trypsin digestion

Prior to use, **9-GN** was purified by HPLC (eluent A 0.10% TFA in water, eluent B 0.10% TFA in CH_3CN, C18 XBridge BEH 300Å 3.5 µm 2.1×150 mm column, 50 °C, gradient 0-70% B in 30 min, 1 mL min^{-1}, detection at 250 nm).

The HPLC fraction containing the folded protein was collected, dried under vacuum and the residue was redissolved in a solution of trypsin (5 µL, 50 ng/µL in 20 mM NH_4HCO_3). The digestion was carried out at 37 °C and the progress of the enzymatic reaction was monitored by MALDI-TOF mass spectrometry (Figure S 26).

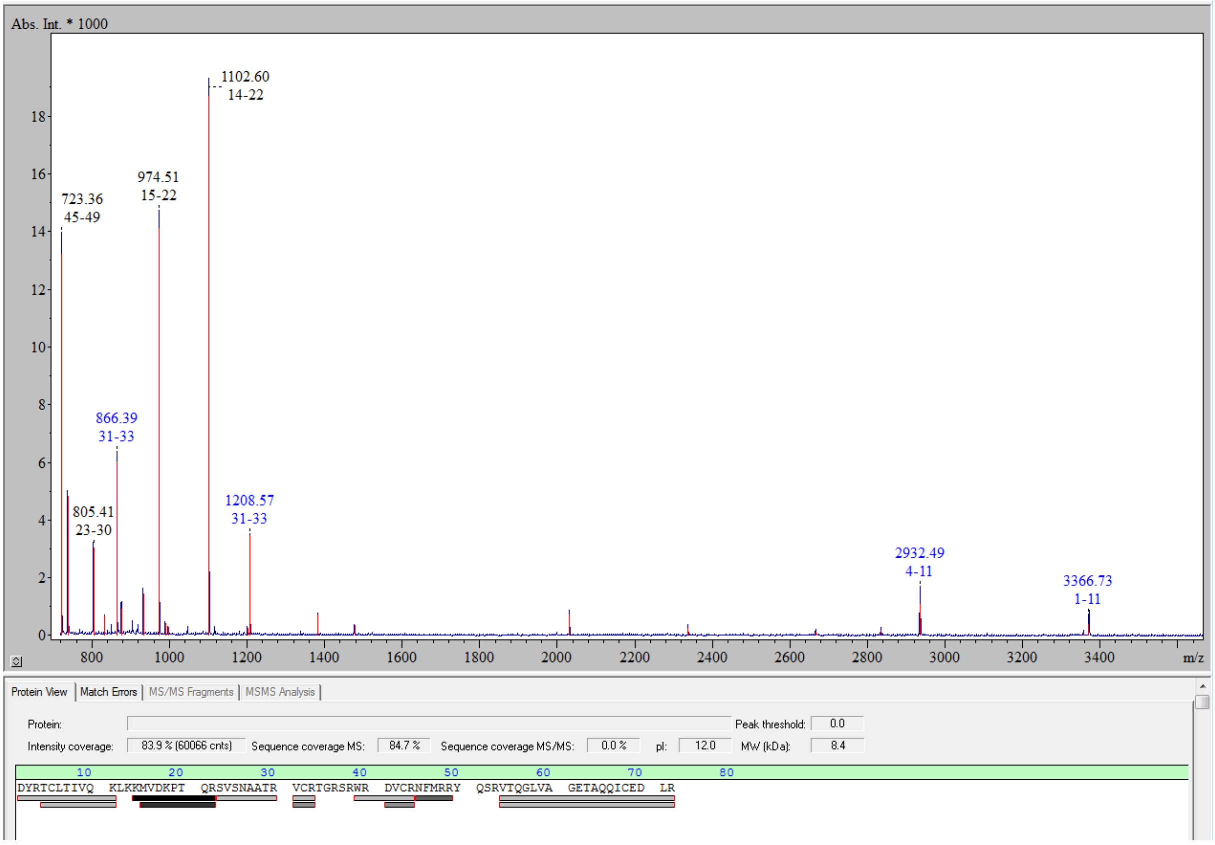

1102.60	1102.60	1.09	14 - 22	1	KMVDKPTQR
974.51	974.51	-0.67	15 - 22	0	MVDKPTQR
805.41	805.42	-9.27	23 - 30	0	SVSNAATR
723.36	723.37	-13.47	45 - 49	1	NFMRR
3366.73	3366.71	3.35	1 - 11	1	DYRTCLTIVQK 5: Crosslink1To[VTQGLVAGETAQQICEDLR]
3366.73	3366.71	3.35	54 - 72	0	VTQGLVAGETAQQICEDLR 15: Crosslink5To[DYRTCLTIVQK] CT: Amidated (C-term)
2932.49	2932.52	-10.07	4 - 11	0	TCLTIVQK 2: Crosslink1To[VTQGLVAGETAQQICEDLR]
2932.49	2932.52	-10.07	54 - 72	0	VTQGLVAGETAQQICEDLR 15: Crosslink3To[TCLTIVQK] CT: Amidated (C-term)
1208.57	1208.58	-5.76	31 - 33	0	VCR 2: Crosslink7To[WRDVCR]
1208.57	1208.58	-5.76	39 - 44	1	WRDVCR 5: Crosslink9To[VCR]
866.39	866.40	-3.40	31 - 33	0	VCR 2: Crosslink6To[DVCR]
866.39	866.40	-3.40	41 - 44	0	DVCR 3: Crosslink9To[VCR]

Figure S 26. MALDI-TOF analysis of the trypsic digest of **9-GN**. Matrix 2,5-dihydroxybenzoic acid (DHB), positive detection mode.VC^{96}R linked to DVC^{107}R by disulfide bridge, calcd. (monoisotopic) [M+H^{+}] 866.40, found 866.39; VC^{96}R linked to WRDVC^{107}R by disulfide bridge, calcd. (monoisotopic) [M+H^{+}] 1208.58, found 1208.57; TC^{69}LTIVQK linked to VTQGLVAGETAQQIC^{132}EDLR by disulfide bridge, calcd. (monoisotopic) [M+H^{+}] 2932.52, found 2932.49; DYRTC^{69}LTIVQK linked to VTQGLVAGETAQQIC^{132}EDLR by disulfide bridge, calcd. (monoisotopic) [M+H^{+}] 3366.71, found 3366.73.

4. Boyden-type microchamber chemotaxis assay

Table S 3. Results of the Boyden micro chamber assay.

	RPMI	MCP-1 (23 nM)	9-GN (nM) 0	1	10	100
**Donor 1**	1.00	8.49	1.87	14.73	17.98	30.46
**Donor 2**	1.00	3.38	1.83	1.33	0.90	2.45
**Donor 3**	1.00	7.22	1.54	1.80	1.56	2.38
**Donor 4**	1.00	55.22	3.03	2.02	4.71	1.68
**Donor 5**	1.00	62.65	14.86	49.20	66.47	76.51
**Donor 6**	1.00	3.75	0.89	1.70	2.51	1.82
**Mean migration index**	1.00	23.45	4.00	11.80	15.69	19.22
**SEM**	0.00	11.29	2.19	7.78	10.48	12.36
